# Raman Spectroscopy Imaging of Exceptional Electronic Properties in Epitaxial Graphene Grown on SiC

**DOI:** 10.3390/nano10112234

**Published:** 2020-11-11

**Authors:** A. Ben Gouider Trabelsi, F. V. Kusmartsev, A. Kusmartseva, F. H. Alkallas, S. AlFaify, Mohd Shkir

**Affiliations:** 1 Department of Physics, College of Science, Princess Nourah bint Abdulrahman University, Riyadh PO Box 84428, Saudi Arabia; amira.bengouider@gmail.com; 2 Department of Physics, Loughborough University, Loughborough LE11 3TU, UK; F.Kusmartsev@lboro.ac.uk (F.V.K.); A.Kusmartseva@lboro.ac.uk (A.K.); 3 Micro/Nano Fabrication Laboratory, Microsystem & Terahertz Research Centre of CAEP, Chengdu, China; 4 Department of Physics, Faculty of Sciences, King Khalid University, Abha PO Box 61421, Saudi Arabia; saalfaify@kku.edu.sa (S.A.); shkirphysics@gmail.com (M.S.)

**Keywords:** epitaxial graphene, SiC, SiC polarity effect, Raman, bubbles, graphene-SiC interface system, density of charge, LOOPC SiC substrate mode, capacitor

## Abstract

Graphene distinctive electronic and optical properties have sparked intense interest throughout the scientific community bringing innovation and progress to many sectors of academia and industry. Graphene manufacturing has rapidly evolved since its discovery in 2004. The diverse growth methods of graphene have many comparative advantages in terms of size, shape, quality and cost. Specifically, epitaxial graphene is thermally grown on a silicon carbide (SiC) substrate. This type of graphene is unique due to its coexistence with the SiC underneath which makes the process of transferring graphene layers for devices manufacturing simple and robust. Raman analysis is a sensitive technique extensively used to explore nanocarbon material properties. Indeed, this method has been widely used in graphene studies in fundamental research and application fields. We review the principal Raman scattering processes in SiC substrate and demonstrate epitaxial graphene growth. We have identified the Raman bands signature of graphene for different layers number. The method could be readily adopted to characterize structural and exceptional electrical properties for various epitaxial graphene systems. Particularly, the variation of the charge carrier concentration in epitaxial graphene of different shapes and layers number have been precisely imaged. By comparing the intensity ratio of 2D line and G line—“I_2D_/I_G_”—the density of charge across the graphene layers could be monitored. The obtained results were compared to previous electrical measurements. The substrate longitudinal optical phonon coupling “LOOPC” modes have also been examined for several epitaxial graphene layers. The LOOPC of the SiC substrate shows a precise map of the density of charge in epitaxial graphene systems for different graphene layers number. Correlations between the density of charge and particular graphene layer shape such as bubbles have been determined. All experimental probes show a high degree of consistency and efficiency. Our combined studies have revealed novel capacitor effect in diverse epitaxial graphene system. The SiC substrate self-compensates the graphene layer charge without any external doping. We have observed a new density of charge at the graphene—substrate interface. The located capacitor effects at epitaxial graphene-substrate interfaces give rise to an unexpected mini gap in graphene band structure.

## 1. Introduction

Graphene is a promising new material emerging from simple sp^2^ bonded carbon atoms arranged in honeycomb lattices. It is considered a unique two-dimensional (2D) carbon allotrope. Theoretical studies of graphene date back to 1947 [1], however, it was not successfully grown until 2004 [2,3]. A 2D system cannot exist in isolation due to their non-thermodynamic stability. The presence of thermal fluctuations at any temperature above absolute zero will destroy any order at greater distances. Therefore, the existence of any 2D structures, such as graphene, is energetically unfavorable. Nonetheless, theory does not exclude the possibility of generating a quasi 2D system in three-dimensional space [4,5,6]. Hence, the presence of a substrate or spatial deformations will stabilize the graphene (or any other 2D) structure. Due to its unique electronic and physical properties graphene and graphene-based materials are intensely studied and are at the forefront of scientific advances and discoveries. For 2D materials, such as graphene, growth and fabrication techniques are vastly important. For example, changes in the stacking order of the graphene layer may be correlated to its size and shape. Understanding the differences that arise in graphene quality and properties as a result of different growth methods is crucial. Initially, exfoliated graphene growth was obtained by a simple exfoliation of single layer graphene from high oriented pyrolytic graphite (HOPG) [2,3]. This method was established by W.A. de Heer, C. Berger and Ph. First team in 2004 [7,8]. Subsequently, in 2007, Chemical Vapor Deposition (CVD) graphene was finally grown. On the other hand, the graphitization process from silicon carbide (SiC) substrate, the so-called epitaxial growth dates back to as early as 1965. Badami et al. describe in detail the formation of carbon planes by heating a SiC surface [9]. Over time this technique has evolved into contemporary graphitization approaches [10]. These are the three main growth methods for producing graphene systems. Additional chemical methods also exist that involve graphene oxidation, reduction of graphene oxide and other organic processes.

Numerous underlying factors influence the exceptional emergent qualities in graphene nano-based materials. Stacking order between the graphene layers (for instance Bernal stacking ABA and AAA) is extremely important and significantly alters the characteristics of graphene-based systems. Manipulating the dimension of graphene containing structures, by designing zero-dimensional (0D) fullerene balls or one-dimensional (1D) nanoribbons [11], (2D) nanotubes [12,13], (3D) newly synthesized 3D graphene [14] and deformed Kirigami and Origami graphene [15] equally dramatically changes the observed behaviors. Similarly, reshaping the graphene layer into advanced forms such us bubbles and domes reveals additional exotic electronic phases [16]. On other hands, it remains still extremely challenging to grow single layer graphene of any appreciable size, despite sophisticate growth control methods. Thus, multilayer graphene is the most commonly obtained systems where graphene layers grow with a vertical stacking order. Multilayer graphene is assigned usually to a bilayer or a tri-layer and normally does not exceed six layers. These layers are bonded with van der Waals bonds [17,18]. The existence of multilayer graphene is critical to understanding the fundamental nature of the electronic states in 2D materials. For example, multilayer graphene demonstrates a metal to semiconductor phase transition and opening up of a gap in the electronic spectrum as the layer numbers are increased [19,20,21,22]. The importance of tuning 2D systems are central in design and fabrication of devices facilitating innovations and advances in applications [23,24].

Graphene demonstrates diverse, exotic electronic singularities, for instance: anomalous quantum Hall effect [25,26,27]. The main reason for these behaviors is that the electrons in graphene behave as massless Dirac-Fermions [16,17,18,19,20,21,22,23,24,25,26,27,28]. The Dirac electrons in graphene demonstrate high charge mobilities that exceed 100,000 cm^2^/Vs to reach maximum velocities of 5 × 10^7^ cm^−2^ s^−1^ [29,30,31]. This is a direct consequence of the Dirac electron spectrum [25] and its extensive optical absorption properties across the terahertz to ultraviolet frequency range. Additional intriguing physical properties in graphene include considerable current densities ~10^9^ A/cm^2^ as well as extremely large thermal conductivities of up to ~5000 W m^−1^ K^−1^ [32]. The carrier density has been shown to be highly susceptible to applied electric field [2]. Notable electric field can be induced by reshaping a graphene layer to form bubbles and domes—accompanied by a complex charge redistribution. The interfaces between such bubble and substrate (in the case of epitaxial graphene SiC) display unique capacitance effects which lead to the emergence of a mini gap in the electronic band structure of graphene of approximately ⁓5 meV. Therefore, the potential of graphene and graphene containing materials for future applications is immense and already shows promise in fields like nano-electronics [16,17,18,19,20,21,22,23,24,25,26,27,28,29,30,31,32,33,34,35,36,37,38,39], nonlinear optics [40] and plasmonics [41].

Graphene nano-electronic applications show a certain degree of dependence on the growth technique. Epitaxial graphene thermally grown from SiC substrate is widely used in electronics. Several main nanoelectronics applications can be identified for epitaxial graphene: Graphene FET (field-effect transistor) for post Si-based complementary metal-oxide-semiconductor (CMOS) applications; transistors, integrated circuit and sensors. Indeed, epitaxial graphene is most frequently used in field effect transistors applications. What makes graphene an excellent material for FETs is its high carrier mobility and nano-scale thickness. The combination of these properties ensures a dissipation less motion of charges along the graphene channel. The transfer of the injected carriers is governed by a gate voltage that induces an electric field. The application of a negative gate bias increases the charges energy, while a positive gate bias has the opposite effect. The gate voltage also influences the electronic structure of graphene—its charge density, Fermi energy and the associated density of states. The nature of the local charge carriers is determined by the location of the Dirac point with respect to the Fermi energy. When the Dirac point is below the Fermi energy, the dominant conduction is via electrons. The opposite scenario produces hole carriers. The limitations of a graphene FETs come from the gapless nature of graphene. As a result, it is impossible to switch off completely a graphene-based FET compared to its semiconductor counterpart [42,43]. Therefore, large number of studies try to modify graphene layer properties in order to induce the opening of a gap. Some of the more common approaches to achieve this include formation of graphene ribbons [44,45,46,47,48,49,50,51,52,53], coexistence of various graphene layer numbers in single system [54], reshaping of graphene layer into bubbles and domes [16]. Epitaxial graphene is also frequently used in radio-frequency (RF)-transistors and amplifiers devices. This is due to its high carrier mobility, unusual transconductance and its nanoscale. Successful implementation of graphene in RF devices has been widely demonstrated throughout the scientific community [35,46,55]. The application of epitaxial graphene in wafer scale integrated circuits (IC) and other graphene-containing devices shows equally exceptional promise [56]. On other hand, designing magnetic field detectors is extremely challenging because of factors related to sensitivity, linearity, stability, size and range. Nevertheless, epitaxial graphene exhibits many characteristics such as anomalous Hall effect [57], high carrier mobility and narrow dimension (an atomic layer dimension) which make it competitive and viable as a magnetic detector. Multiple Hall effect-based nano-scale magnetic field detectors have been successfully realized [58,59], particularly, Hall sensors [60]. Additional applications of epitaxial graphene include FET detectors [56], Terahertz detector [61] and UV detectors. Epitaxial graphene, due to its organic C-based origin, has also many uses as a biosensor, molecules and gas sensor. The adhesion of molecules onto graphene induces local charge concentration variations which are readily detectable. This sensitivity is related to graphene’s unique charge density concentration at the neutrality point that is highly susceptible to slight perturbations in the chemical potential though chemical gating and impurity doping. Therefore, minute variations of the Fermi level may induce substantial changes in the carrier concentration. Adsorbing molecules may also donate or deplete electrons. The most prominent applications of graphene as molecular detectors include NO_2_ sensors [62]; diagnostic biosensors of human chorionic gonadotropin (HCG) that are used as markers in pregnancy and different cancer therapies [63].

Designing new 2D materials requires a powerful analysis tool to investigate their fundamental properties [64,65,66]. Raman spectroscopy has been established as a reliable technique to probe and characterize carbon-containing materials and especially, graphene. Raman spectroscopy is considered as a non-destructive and non-invasive method to examine the physical and electrical characteristics of graphene materials [66,67]. The approach may address graphene’s structural properties and defects, lattice strain, the density of charge, layer numbers, as well as the stacking order of the layers [68,69,70,71,72,73]. Raman spectroscopy identifies the main fingerprints of graphitic materials such as D, G, 2D, G* and 2D’ modes. A typical signature of epitaxial graphene shows two main lines known as the G and D modes appearing around 1580 and 1360 cm^−1^, respectively. The G mode is assigned to the E_2g_ phonons at the Brillouin zone (BZ) center (the Γ-point). The D-band is only activated through an intervalley scattering between defects and (TO) phonon modes at K-point. The D-band images actively lattice distortions and other local defects [74,75]. The 2D-band is associated with two phonon modes. In the case of monolayer graphene this mode appears as a single band, while for a bilayer it is decomposed into four Lorentzian bands. The 2D mode becomes broader as the layer number is increased. Specifically, by tracking the changes in the 2D band important information related to graphene quality could be obtained. Additional weaker modes like D’ and 2D’ bands appearing respectively at 1620 cm^−1^ and 3240 cm^−1^ are also of interest [65]. The D′ mode is activated only by an intravalley process, where a transition of the scattered electron occurs between two points at K (or K’) in the BZ. While, 2D’ Raman mode is related to an intervalley scattering.

In this work, we will review the principal Raman scattering processes of SiC substrate in both polarities and explain epitaxial graphene growth. We will introduce the epitaxial graphene Raman signature. We will describe different approaches addressed to identify graphene layer number and characterize electrical properties in various epitaxial graphene systems. Particularly, we will highlight the correlation between the density of charge and the different shapes and layers number in epitaxial graphene. The role of the SiC substrate will also be addressed. The density of charge will be obtained via the investigation of the longitudinal optical phonon coupling (LOOPC) mode of the substrate. Graphene layers number will be established by following the mode intensity ratio I_G_/I_2D_. Our findings demonstrate that by comparing the local density of charge in epitaxial graphene to its shape and number of layers we have discovered new and unexpected capacitance effects. The substrate (SiC) self-compensates the charge in the graphene layer without any external doping. The result is a unique and unusual density of charge at the substrate-graphene interface. 

## 2. Silicon Carbide Substrate Properties Directed to Epitaxial Graphene Growth

Silicon carbide ‘SiC’ substrate was initially grown by Jacob Berzelius in 1824 [76]. This substrate exhibits exceptional thermal, structural and electrical properties compared to other semi-conductors. The SiC substrate possesses: a small lattice constant, considerable band gap E_g_, elevated melting point, resistance to oxidation and erosion, low thermal expansion coefficient and excellent chemical stability, which make it highly attractive in nanoelectronics for device fabrication [77,78,79]. These properties mainly originate from its structure which consists of covalent bonds between the silicon and carbon atoms covering 88% of the lattice [80]. Prior to successful SiC growth such covalent bond density was very difficult to produce in the lab [80]. Its structure also imposes a polarity effect that depends on the Si and C termination order at materials surface. The polarization significantly affects the observed physical and chemical properties. SiC is allotropic, displaying multiple polytypes [81]. The ability to control the properties, by combining the polarity effects due to surface termination with an appropriate choice of the polytype makes SiC an ideal candidate substrate for future electronics.

### 2.1. Structural Properties of Silicon Carbide Polytypes

**Figure 1:**
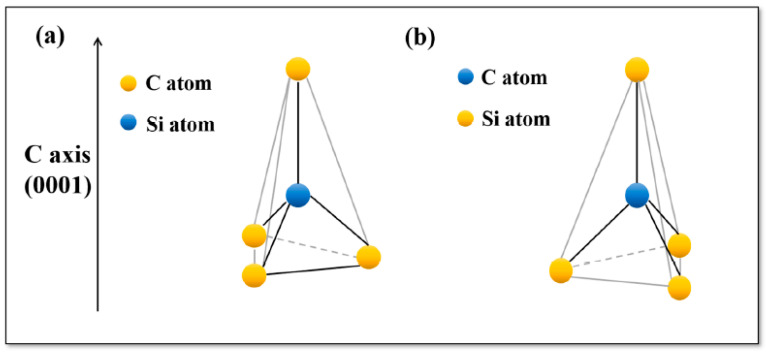
Elongated tetrahedron with C_3v_ symmetry of the hexagonal polytype: (**a**)—SiC_4_ each silicon atom is bonded to 4 carbon atoms and (**b**)—CSi_4_ each carbon atom is bonded to 4 silicon atoms.

Silicon carbide (SiC) crystallizes in different forms defined by various polytypes [77,81]. All these allotropes demonstrate a compact hexagonal arrangement of silicon (carbon) atoms, where the halves of the tetrahedron sites are often occupied by carbon (silicon) atoms. Various SiC tetrahedron symmetries exist. For instance, in the only cubic polytype (3C-SiC) the tetrahedral symmetry is T_d_. While, the elongated tetrahedron configurations associated with the most common hexagonal polytypes (4H and 6H-SiC) have the C_3v_ symmetry. The two possible elongated configurations of the tetrahedron SiC_4_ and CSi_4_ arising in hexagonal SiC polytypes are shown in Figure 1. Here, each carbon (silicon) atom is surrounded by 4 silicon (carbon) atoms. The bonds linking silicon and carbon atoms are solid bonds, where the tetrahedron stacking may turn up to 60° without disturbing the crystal continuity along c axis. These bonds are a covalent bond of sp^3^ type. On the other hand, the electronegativity difference existing between the SiC atoms increases the contribution of the ionic energy into the bond by almost 12% as estimated using Pauling’s formula [80]. This small positive charge on the Si atom gives a slight ionic character to the interatomic bonds. 

**Figure 2:**
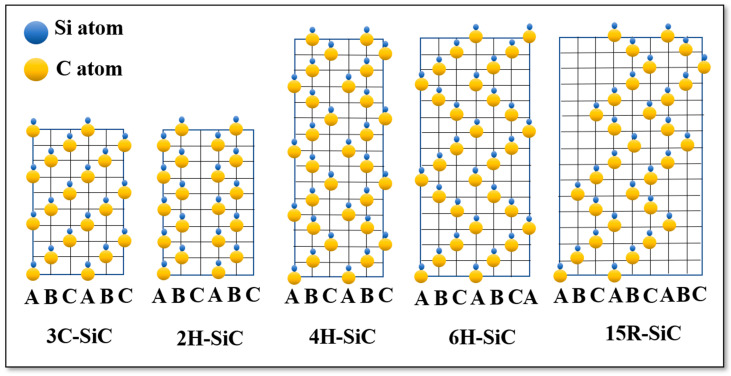
Stacking Si-C bilayer in the plane (11$\bar{2}$0) along the vertical axis (0001) for the most common polytypes, where the atomic plane positions in each polytype are described as A, B and C. The modification in the direction from right to left in the stack corresponds to the intercalation of an opposite tetrahedron in relation to those of the lower bilayer.

SiC polytypes could be identified through the stacking order of Si-C bilayer. The cubic structure 3C-SiC of trigonal symmetry is composed of simple stacking of the same tetrahedron SiC_4_ (CSi_4_) along c-axis (see, Figure 2). Whereas, the hexagonal structure consists of simple alternation of both possible configurations of the tetrahedron along c-axis (see, Figure 2). Ott and al. considered the crystallographic changes of SiC as equal structures, where all symmetries represent an identical layer stacked perpendicular to the hexagonal or trigonal axes. Indeed, trigonal or hexagonal symmetry could be described based on the common hexagonal axis equivalent that arises perpendicular to each one of the normal planes (a, b and d) and by forming a 120° between them. The separation distance between the carbon plane to the neighboring silicon planes represents almost 1/3 of the ratio compared to the carbon-carbon interplanar distance. This induces a polar behavior along the symmetry axis perpendicular to the carbon planes, despite the infinite crystalline structure remaining in between these two limit structures. The polar periodicity arises when one type of tetrahedron (SiC_4_) is replaced by another type (CSi_4_) along the c axis. The complexity originating from the numerous possible stacking sequences of the tetrahedra results in approximately 250 different polytypes [82].

This large variety of the existing SiC polytypes gives rise to vastly different electronic properties. The SiC band gap varies substantially from one polytype to another. The band gap in 3C-SiC is 2.390 eV, while the band gap in 4H-SiC and 6H-SiC is 3.263 and 3.023 eV respectively [83]. Due to its narrow band gap and large isotropic electron Hall mobility the 3C-SiC is extremely promising compared to the other polytypes [83]. However, the hexagonal polytypes (4H and 6H-SiC) are the most commonly encountered structure types, due to higher stability at elevated temperatures ~1700 °C. Another unique property of these polytypes is the different termination sequences of the Si-C bilayer. Depending on the overall pattern, the bilayer can terminate either with a C or a Si atom at any one location. The observed polarity and the presence of a polar axis in SiC is firmly linked to the order and orientation of the atoms at the surface. The polarization is attributed to the different energies of the SiC atoms that terminate at a particular surface site of the lattice [84,85].

### 2.2. Raman Signature of SiC Substrate

**Figure 3:**
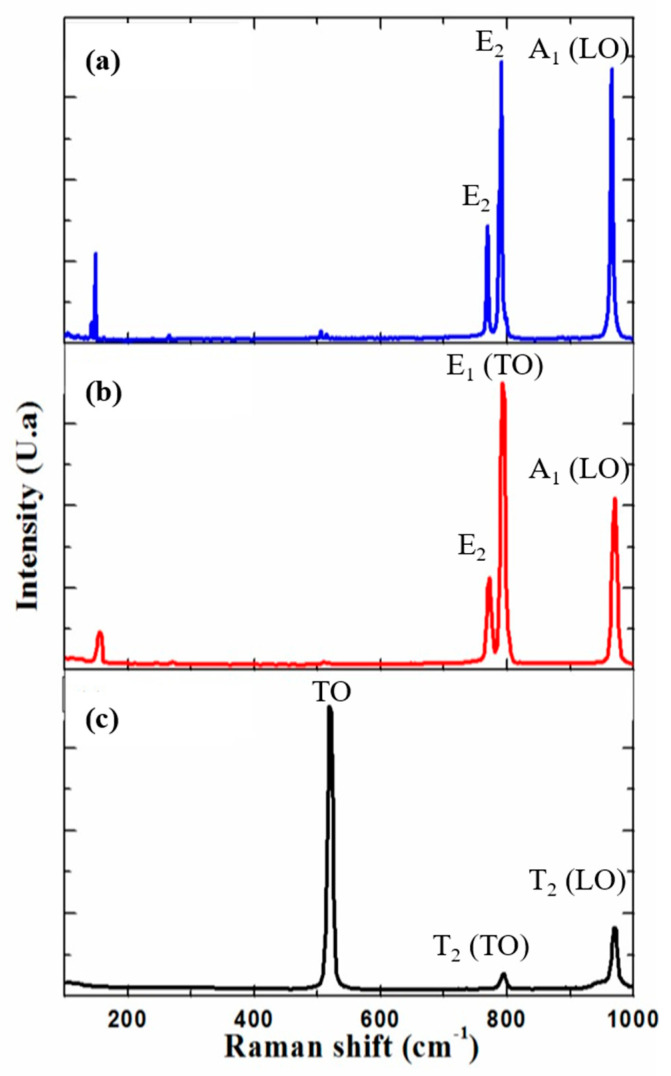
Raman signature spectra for the SiC polytypes: (**a**)—6H (0001)-SiC, (**b**)—4H-SiC (000-1), (**c**)—3C-SiC (100)/Si (100). The main Raman modes are indicated. The strong peak at 500 cm^−1^ in (**a**) corresponds to the TO (Γ) vibrations in the Silicon wafer Si (100).

Various SiC polytypes were investigated during the epitaxial growth of graphene layers, for example: 3C, 4H- and 6H- SiC polytypes. However, the hexagonal polytypes are most commonly used for graphene growth. The hexagonal polytypes of SiC are denoted as nH, where n refers to the number of SiC bilayer present in the unit cell (n = n _SiC bilayer_). The dispersion curves of SiC polytypes are related to each other. The division of the Brillouin zone of 3C-SiC by n _SiC bilayer_ gives rise to the dispersion curves for the different hexagonal polytypes. The splitting of the Brillouin zone, when passing from one polytype to another, is associate with additional new Raman modes. Each polytype has a unique Raman spectrum that can distinguish it from the others. The Raman spectrum for the most common SiC substrates shows various active modes between 100 cm^−1^ and 1000 cm^−1^ (see Figure 3). Figure 3a represents the Raman spectrum for the 6H-SiC (0001) substrate. Main Raman lines have been found between 760 cm^−1^ and 970 cm^−1^. We have located E_2_, E_2_, E_1_ (TO) and A_1_ (LO) around 765 cm^−1^, 785 cm^−1^, 795 cm^−1^ and 965 cm^−1^, respectively [85,86,87]. Figure 3b shows a typical Raman spectrum of 4H-SiC (000-1). Four main Raman lines have been distinguished between 250 cm^−1^ and 970 cm^−1^. We have observed E_1_ (low) at 270 cm^−1^, while the E_2_, E_1_ (TO) and A_1_ (LO) lines appeared respectively around 770 cm^−1^, 790 cm^−1^ and 965 cm^−1^. Figure 3c shows a typical spectrum for 3C-SiC (100)/Si (100). Mainly, two Raman modes were located approximatively around 796 cm^−1^ and 972 cm^−1^ corresponding, respectively, to the T_2_ (TO) and T_2_ (LO) lines [83]. The additional mode occurring at 521 cm^−1^ is related to vibrations in the Silicon wafer TO (Γ).

### 2.3. Polar Behaviour in SiC Substrate

**Figure 4:**
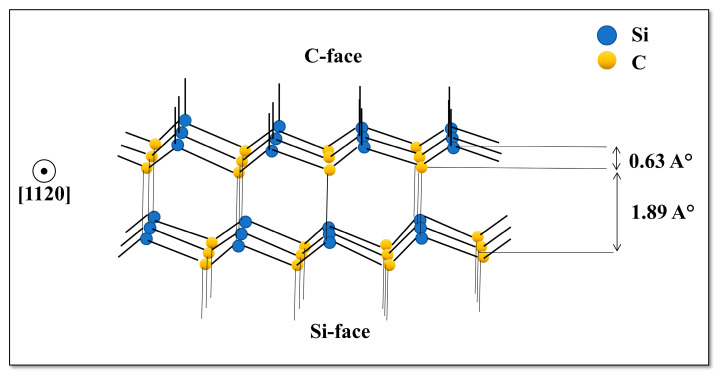
Schematic representation for the two different Si and C termination faces of the hexagonal polytype of SiC crystals, with orientation (0001) and (000-1), respectively. Si atoms are shown in blue, C atoms are shown in yellow. The C terminated face is located at the top and the Si terminated face is located at the bottom of the structure diagram.

The presence of a polar axis in SiC substrate is related to face termination and leads to many unique properties [86]. SiC planes are constructed by stacking identical close-packed double layers of silicon and carbon atoms., where the dominant separation distance occurs between atoms of the same type i. e carbon-carbon (or silicon—silicon) arranged in a tetrahedron (Figure 4). In each tetrahedron a Si atom is bonded to four C atoms or vice versa a C atom is surrounded by four Si atoms. The bond distances binding the Si (C) atom to the 3 base C (Si) atoms are smaller than the Si-C interplanar bond length. The Si-C interplanar bond length is also smaller than the monoatomic interplanar distance C-C (or Si-Si). This particular crystallographic configuration encourages the SiC to split into two distinct (faces)—the C face and the Si face, chemically different in nature (as shown in Figure 4). These two opposites faces of the crystal are perpendicular to the polar axis. The Si terminated face is labelled (0001) and the C terminated face is labelled (000-1), respectively [88]. The [0001] vector has the same direction as c-axis, where it points from the C face to the Si face. The polar axes are <0001> and <111> for α-SiC and β-SiC polytypes respectively. The face termination is defined relative to the polar axis. Thus, the face terminated with Si is labelled as (0001) for α-SiC and (111) for the β-SiC. While, the face terminated with C is labelled as (000$\bar{1}$) for the α-SiC and ($\bar{1}\;\bar{1}\;\bar{1}$) for β-SiC. The two face terminations significantly affect the polarities, physical and chemical properties as well as growth processes. In particular, growth processes occur faster on face terminated with carbon than the face terminated with silicon [80,81,82,83,84,85,86,87,88,89,90]. The surface energy variation (1.76 × 10^−4 J^/cm^2^ for the Si-face and 0.71 × 10^−4 J^/cm^2^ for the C-face) remains the main factor for the observed discrepancy [29]. The face termination also affects the electrical properties, impurity intercalation and oxidation kinetics. 

## 3. Epitaxial Graphene Growth Dependence on the Substrate Polarities

Epitaxial graphene was grown by Walt de Heer in Atlanta in 2004. Earlier studies demonstrate SiC substrate graphitization under high temperature [9,10]. In general, epitaxial growth exploits the similarity and symmetry between lattices of different crystals. Epitaxial graphene refers to graphene layers grown from a silicon carbide (SiC) substrate. In this case, two crystals are grown in parallel—graphene and SiC—with different relative growth rates, resulting in epitaxial graphene. The lattice constants of graphene and SiC are closely matched which makes epitaxial growth viable. Epitaxial graphene shows high thermal stability (up to ≥1000 °C) [91,92,93]. The graphitization of SiC occurs in a particular temperature range which depends on the Si or C face termination [93,94,95]. The process begins by sublimation of silicon atoms from the Si-C bilayer. The sublimation temperature for Si atoms is less than 1000 °C which is lower than that of carbon atoms. The graphitization occurs after silicon atoms from three consecutive Si-C bilayers have sublimated, eventually forming a single layer of graphene. During graphitization carbon atoms of the remaining three Si sublimated bilayers rearrange into graphene honeycomb lattice. The density of carbon contained in three SiC bilayers is identical to that found in a graphene layer. However, many different atomic phases are observed at the beginning of graphitization [82,83,84,85,86,87,88,89,90,91,92,93,94,95]. Experimentally, graphitization can occur under ultra-high vacuum “UHV” using various flux like Si and Ar. For UHV, epitaxial graphene is grown at pressures less than 10^−9^ mbar under heating induced by electron bombardment or in an annealing furnace under a secondary vacuum (10^−5^ mbar). It important to note, that growing epitaxial graphene using Si and Ar flux results in higher graphene quality compared to the UHV method. In particular, larger graphene flakes can be grown using the flux method. 

### 3.1. Si-Face Grown Epitaxial Graphene 

**Figure 5:**
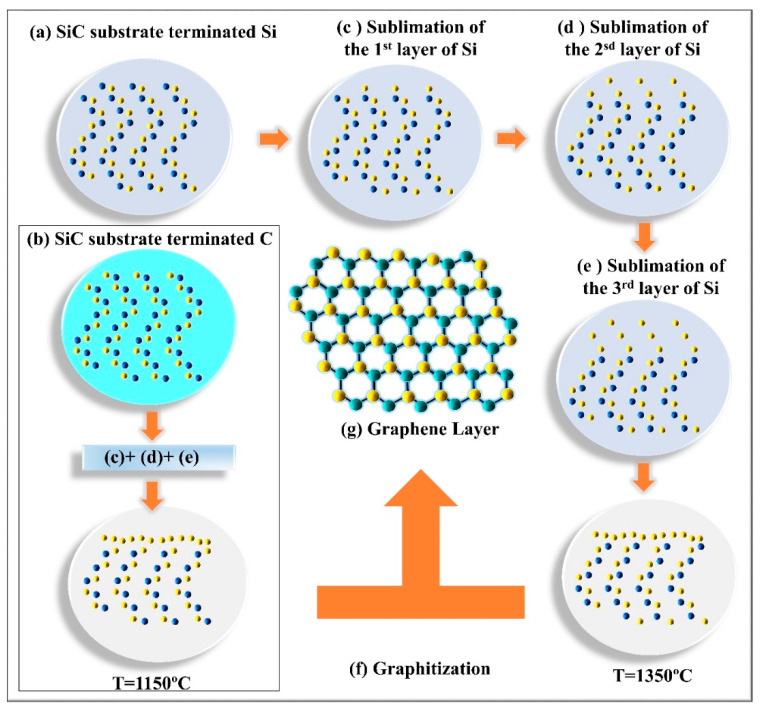
Epitaxial graphene growth on SiC substrate: (**a**)—face terminated silicon (Si), (**b**)—face terminated C, (**c**)—sublimation of the first Si layer, (**d**)—sublimation of the second Si layer, (**e**)—sublimation of the third Si layer, (**f**)—graphitization occurring at T = 1350 °C for the Si-terminated face and at T = 1150 °C for the C-terminated face, (**g**)—graphene layer formation.

Epitaxial graphene grown on face terminated Si displays a particular growth process compared to that of face terminated carbon. Here, a unique graphitization phase was observed before the formation of the first graphene layer [35]. This phase strongly depends on the annealing temperature where it was found at 1150 °C. It has C-atoms density close to that of graphene. Its diffraction pattern shows a unit cell equal to about 6√3 × 6√3R30 of the graphene cell. This phase is called the buffer layer or zero layer and is labelled as 6R3-SiC. In comparison, the unit cell of SiC face terminated Si is equal to a 13 × 13 graphene unit cell [82,94,96]. Epitaxial graphene on face terminated silicon occurs under UHV. The first graphene layers appear at temperatures up to 1350 °C (see, Figure 5a). The process is gradual going through the buffer layer [97,98,99]. The presence of a buffer layer imposes a high growth order, similar to graphite. Thus, epitaxial graphene on SiC face terminated Si produces large, good quality flakes reaching 100 nm in size—the quality surpasses that of exfoliated graphene. 

The high quality of this type of epitaxial graphene allows to explore in detail its new and unconventional electronic properties. Ideal graphene has unique band structure defined by the presence of a “Dirac cone” [100]. In epitaxial graphene, The Dirac point is located 0.27 eV below the Fermi level and the carbon layers are doped with electrons. This type of epitaxial graphene shows electronic properties very similar to ideal graphene. Thus, despite the presence of the substrate underneath the graphene layers the electronic structure or the “Dirac cone” is still preserved. The slight differences between epitaxial graphene and ideal theoretical predictions arise because of the normalization of the “Dirac cone” structure due to electron-electron and electron-plasmons interactions [45]. In addition, electronic abnormalities result from the presence of bilayer-segments with various stacking sequences. Epitaxial graphene shows unusual and exotic transport properties. The system behaves as a two-dimensional electron gas, reaching mobilities ~1100 cm^2^/Vs and charge carrier densities equal to ~10^12^ cm^2^. Epitaxial graphene displays quasiparticle chirality and Landau levels in a magnetic field. However, despite the unusual magneto-transport properties in Si-face grown epitaxial graphene, quantum Hall effect was not observed in this system.

### 3.2. C-Face Grown Epitaxial Graphene

Unlike Si-face grown epitaxial graphene, there are fewer studies interested in the early graphitization stages of the C-face grown epitaxial graphene (SiC (000-1)). The graphitization temperature for C-face grown epitaxial graphene is about 1100 °C (see, Figure 5b). No buffer layer was reported [94,95]. The graphene layer grown on this termination displays rotational disorder between the grains contrary to graphene grown on face terminated Si [96] and generally results in constructed flakes of smaller size. Graphene layers stacking order and their size depend extremely strongly on the conditions of the growth process (e.g., temperature). Similarly, less information is available about the electronic properties of C-face grown epitaxial graphene. To date, a linear band structure, the presence of a Dirac cone, neutral carbon planes and Landau levels in an applied magnetic field have been established. This type of epitaxial graphene has been confirmed to behave as a two-dimensional electron gas by Shubnikov de Haas’ oscillations studies. C-face grown epitaxial graphene demonstrates much higher mobilities ~27,000 cm^2^/Vs and carrier densities of ~1 × 10^12^ cm^−2^. The observed difference between Si-face and C-face grown epitaxial graphene could be attributed to the specifics of the growth method, which impacts the structural properties.

The electron transport properties depend on the graphene-substrate interactions, as a charge transfer occurs between the substrate and the carbon layers. Thus, the graphene layers nearest to the interface are electron doped, virtually decoupled from the substrate and dominate transport. The doping shifts the Fermi energy to 0.21 eV above the Dirac point [28]. The electronic structure of the substrate behaves similarly to multilayer graphene with AB stacking order or to that of graphite and, therefore, has a low impact on electron transport. Epitaxial graphene grown on face terminated C displays better transport properties then that grown on face terminated Si, which may be related to more than an order of magnitude difference in their respective mobilities 27,000 cm^2^/Vs > 1100 cm^2^/Vs. The ability to control the structural and electronic properties of epitaxial graphene by choosing an appropriate SiC polytype substrate and a particular polarity (face-termination) makes this material an excellent, versatile and adaptable candidate for electronic device design and fabrication. The applications range of epitaxial graphene is rapidly expanding.

### 3.3. SiC polarity Effect on the Grown Graphene Layers

The different SiC polarities used in epitaxial graphene growth strongly affect the morphological and electrical properties of the obtained layers. Indeed, the surface quality and the growth process vary according to the face termination as discussed above. Particularly, the Si-face grown graphene demonstrates large dependency on the substrate underneath. This is mainly due to the buffer layer existing at graphene-SiC substrate interface. The buffer layer is often called the zero-layer and corresponds to the first carbon-rich plane of atoms. Its structure, generally denoted as 6R3-SiC differs from single layer graphene. Despite its rich density in carbon atoms, arranged in honeycomb lattices, this layer could not be considered as a graphene layer. This is due to the sp^3^ bonds linking the buffer layer to the Si atoms of the substrate termination. Conversely, in graphene the carbon atoms display sp^2^ bonds. Graphene couples to the surrounding layers, such as the buffer layer and substrate via weak Van der Waals bonds. Whereas, the sp^3^ bonding in the buffer layer induces a much stronger interaction to the substrate. The buffer does not contain π bands found in graphene. For this reason, the electronic properties of the graphene and the buffer layer are dissimilar [101]. The buffer layer presence affects the morphological properties in graphene. Tinny graphene flakes have been located. This problem has been solved through interface and graphene growth from substrate step edges [102]. Such a find ensures a better graphene layer dimension as well as a better quality. On the other hand, the buffer layer existence significantly disturbs the electrical properties. Indeed, such a layer induces donor states that dope graphene [10] and open up a wide band-gap. Here, the associated Fermi level corresponds to a small dispersion state related to the buffer layer-SiC bonds. Therefore, a weak charge density distribution has been found for Si- face terminated graphene compared to freestanding and to C-face grown graphene [10]. For different layers number order, the charge density distribution enormously decreases compared to that of freestanding graphene. This could be ascribed to the strong interaction between the graphene layer and the substrate for single layer graphene case. On the other hand, the increase in the graphene layers number induces a better stacking order that plays main role in controlling the charge density distribution. Here, several symmetries of the graphene layers stacking may arise, which strongly affects the associated band structure and magnetic response. However, the large hampering resulting from such a buffer layer has been resolved using new approaches that is, atoms intercalation at graphene—SiC interface. Nevertheless, it is important also to highlight the advanced electrical properties located on C-face grown graphene. This latest behaves identically to quasi-free-standing graphene, where no buffer layer exists. This explains its good transport properties. However, due to the absence of the buffer layer poor morphological quality distinguished such a kind of graphene layer. Indeed, the buffer layer presence ensure better stacking order of the graphene layers along the c-axis as well as large size of graphene flakes.

### 3.4. Raman Signature of Graphene Layer

The Raman spectrum of ideal graphene layer, i.e., exfoliated graphene, exhibits noticeable features. The main characteristics are the G line and 2D line, generally, observed at around 1580 cm^−1^ and 2700 cm^−1^. G line is assigned to E_2g_ phonons. The 2D line is a second order Raman mode, that is, assigned to two phonons process, of the D band. The 2D band is sometimes labelled as G’ band and has been shown to be related to graphene growth by the double resonance model [65]. Specifically, in epitaxial graphene changes in the FWHM of the 2D line correlate strongly to the graphene layers number (see Table 1). Other low intensity modes are also present in graphene Raman spectrum: G^∗^, D’ and 2 D’ located at 2687 cm^−1^, 1620 cm^−1^ and 3240 cm^−1^ respectively. The G∗ band originates from an intervalley scattering between one TO and one LA phonon, located around two different Dirac cones at K (or K’) points in BZ. The G∗ band is identified purely for single layer graphene and disappears completely with increasing layer numbers [103,104]. The D’ band is assigned to an intravalley scattering, where only transitions on the same Dirac cone are considered. The 2D’ band is the second order process of the D’ band [105,106]. The 2D’ mode does not require an intravalley momentum transfer for its activation.

**Figure 6:**
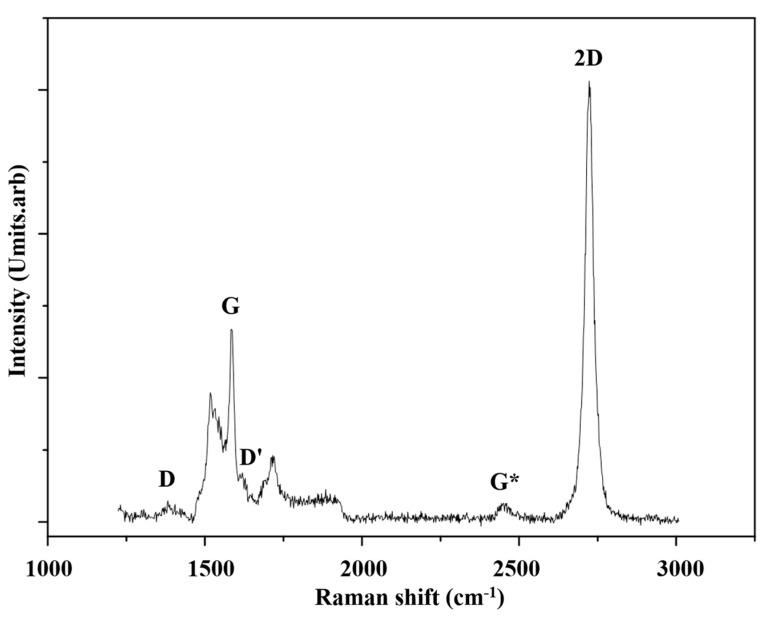
Typical Raman spectrum of a single layer of epitaxial graphene [16].

**Figure 7:**
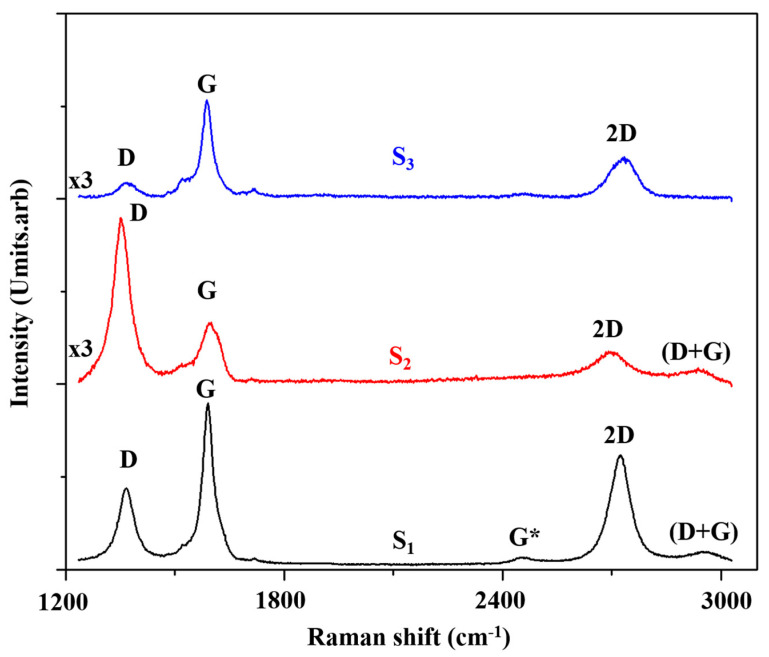
Raman spectra obtained randomly in different zones of three epitaxial graphene samples respectively: S_1_ grown on 4H-SiC (0001), S_2_ grown on 3C-SiC (100)/Si (100) and S_3_ grown on 6H-SiC (0001). Major graphene Raman modes have been located.

The Raman spectrum of epitaxial graphene demonstrates a small number of differences to that of ideal graphene (see, Figure 6). The few signature Raman bands of epitaxial graphene are the D band and the (D + G) band, which do not commonly appear in ideal graphene. The D and (D + G) modes are located at around 1365 cm^−1^ and 2954 cm^−1^, respectively. The D band is induced by disorder and is related to the fields produced by defects or at the edges of the graphene flakes. The D band occurs at about 1360 cm^−1^. The D-peak originates from the breathing of six C atoms forming the hexagon, that is, this corresponds to the transverse optic (TO) phonon arising at the K-point of the BZ. It is activated by an intervalley scattering process and for its activation lattice distortions or a defect is needed [107]. In comparison, the 2D band is completely unrelated to disorder and is simply the second order of the D band, occurring at double the frequency of the D band. A pronounced D band is usually observed in epitaxial graphene (see, Figure 7) contrary to ideal graphene, where it appears only at the edges. This difference arises, because epitaxial growth cannot prevent the existence of defects. The other unique Raman line the so called (D + G) band which occurs at about 2954 cm^−1^. In fact, this Raman mode is only found in epitaxial graphene grown on C-face terminated substrate. Studying the Raman modes can reveal many intrinsic physical and electrical aspects of graphene. The most fundamental property that could be determined by Raman spectroscopy is graphene layers number. 

The most effective approach to determine the number of layers n_EG_ in epitaxial graphene is to combine information from the full width at half maximum (FWHM) and the intensity ratio of the G band on the 2D band “I_G_/I_2D_.” This method can be adapted to all kinds of graphene. Investigations of the precise shape of the 2D band and the I_G_/I_2D_ ratio can be used fairy accurately to establish n_EG_ in the range 1 ≤ n_EG_ ≤ 7. The limit n_EG_ = 7 corresponds to the accepted threshold value that separates multilayer graphene from graphite. It has been shown that the Raman spectrum of multilayer graphene with n > 7 is identical to that of graphite. The shape of the 2D band can be predicted accurately by the double-resonance model which can be used to identify the number of atomic planes present in graphene. Specifically, the deconvolution of the 2D band into elemental bands gives the number of atomic layers [26,103,104,105,106,107,108]. For example, in a bilayer the 2D band is split into four bands—making the 2D band wider [103].

The change in the ratio I_G_/I_2D_ with the increasing number of layers n_EG_ was determined empirically and is used as an independent metric for the verification of the number of layers [103,104,105,106,107,108,109]. Nominally, the I_G_/I_2D_ ratio is less than 0.5 (I_G_/I_2D_ < 0.5) for one layer of graphene (n_EG_ = 1). For bilayers (n_EG_ = 2) the I_G_/I_2D_ falls in between 0.5–1 (0.5 < I_G_/I_2D_ < 1). For graphene multilayers (n_EG_ > 5) the ratio is greater still (I_G_/I_2D_ > 1.8). For example, we have found that the I_G_/I_2D_ for a large epitaxial graphene macro-island formed of four layers (n_EG_ = 4) is about 1.5 (see, Table 1) [103,104]. It is very important to note that for epitaxial growth the FWHM, Raman mode position and intensity may all change due to various factors which are unrelated to layer number. The observed changes in these parameters could be due to laser wavelength, doping, charge distribution, substrate effect, oxidation, graphene layer morphology, strain and growth-induced disorder. We have summarized some of the results for epitaxial graphene in Table 1. 

The Raman spectrum of epitaxial graphene is quite unique because it also contains several second order modes coming from the SiC substrate located in the frequency range [1000–2000 cm^−1^]. Many of these Raman bands coexist with graphene’s G band in the 1450–1750 cm^−1^ interval. For example, the band observed at around 1520 cm^−1^ is the second order mode from the TO(X) phonon located at 762 cm^−1^. Similarly, the band located at 1714 cm^−1^ is assigned to optical phonons around the M-point of the BZ [109]. The hexagonal SiC polytypes show an additional Raman mode at 1542 cm^−1^ coming from the doubling of the phonon symmetry around the M-point at 771 cm^−1^. 6H-SiC displays a band at 1526 cm^−1^ which is related to the phonons of the L-M branch.

## 4. Raman Investigation of Electrical Properties in Epitaxial Graphene

### 4.1. Raman Spectroscopy and Coherence to Electrical Measurements of Graphene 

The electrical properties in graphene layers are established using standard 4-probe transport measurements in a source-drain configuration. The observed electrical properties are significantly affected by doping and other parameters (e.g., stain, flake edges). Raman spectroscopy is a powerful technique to investigate graphene quality and doping [110,111,112,113,114,115,116,117,118,119,120]. Therefore, it is extremely informative to correlate Raman spectra with corresponding electrical behavior in graphene samples. 

As described before, the graphene Raman signature is principally identified through the location of the G and 2D modes, found at 1584 and 2700 cm^−1^ respectively. The G and 2D modes are assigned to different Raman scattering order modes: a first order (G mode) and a second order (2D band). The activation of the 2D mode depends on the energy of the excitation laser [115]. The mode activation width K + ∆k is determined by the double resonances and the linear dispersion of the phonons around K-point [116,121,122]. It is important to highlight that the electrical properties in graphene are strongly correlated to the changes in the 2D band position and character. This was established empirically through multiple experimental works.

The earliest report using Raman spectroscopy to probe the effect of electron and hole doping in graphene showed that the positions of the G and 2D bands as well as the FWHM of the G band change dramatically with carrier concentrations [116]. Specifically, it was found that the Raman shift of the G band behaves symmetrically for equal electron and hole doping percentages around a doping minimum [115]. In addition, it was shown that the G band FWHM is reduced as the electron/hole doping is increased [117,118]. This occurs due to the Pauli exclusion principle that blocks the conversion of phonons into electron–hole pairs as soon as the electron–hole gap becomes larger than the phonon energy. Conversely, the behavior of the 2D band is highly dependent on the nature of local doping and demonstrates different properties in the presence of electron or hole carriers. The 2D band is also far more sensitive to the presence of the dopants than the G band. Therefore, the doping response is most accurately obtained by considering the intensity ratio of the G and 2D peaks (I_2D_/I_G_). The ratio of the peak intensities I_2D_/I_G_ has also been found to represent the density of charge in the sample. The shape of the 2D mode can additionally determine local layers number and effectively identify single layer graphene [115,117,121,123]. Raman analysis of both G and 2D modes is an established approach to characterize the electrical properties in epitaxial graphene. However, recent work by Amira et al. introduces a completely new method to probe the electrical properties of epitaxial graphene using substrate Raman modes. The results of the Raman spectroscopy studies are consistent and show good agreement with the electrical transport experiments in epitaxial graphene.

### 4.2. Graphene Raman Modes Locating Electrical Properties Changes

We have performed a Raman spectroscopy study for three epitaxial graphene—S_1_, S_2_ and S_3_ grown on three different SiC substrates. Sample S_2_ was grown using 3C-SiC (100)/Si (100) substrate. Samples S_1_ and S_3_ were grown respectively on C face terminated 4H-SiC (denoted 4H-SiC (000-1) and also 4H-SiC (000$\bar{1})$) and Si face terminated 6H-SiC (denoted 6H-SiC (0001)). All samples showed active Raman peaks between 1000 cm^−1^ and 3000 cm^−1^ (see, Figure 7). Representative spectra for S_1_, S_2_ and S_3_ are shown in Figure 7. The spectra contained contributions from the second order Raman bands of the SiC substrate as well as the second order Raman modes of graphene. The SiC substrate modes were mostly found at around 1470 cm^−1^ and 1910 cm^−1^ [107]. Major epitaxial graphene lines such as: D band, G band and 2D band have been located. The results were analyzed by comparing the intensity ratio I_2D_/I_G_ with the Raman shift position of the 2D band and its FWHM. After identifying areas of single layer graphene, we have investigated the intensity ratio I_2D_/I_G_ ratio and its dependence on the various substrate polytype. Studies were conducted on different surface areas to eliminate random errors. The Raman spectra have shown a graphene flake distribution across sample surfaces.

**Figure 8:**
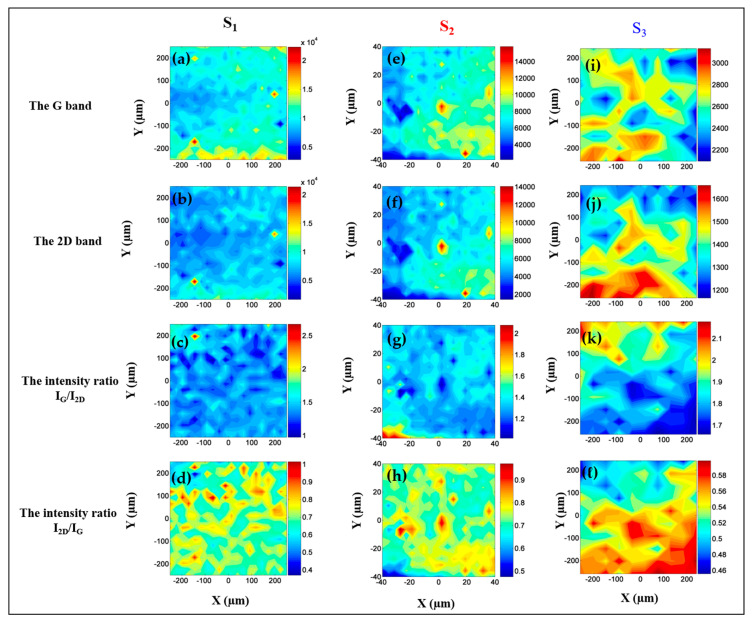
Raman intensity maps for the three epitaxial graphene samples S_1_, S_2_ and S_3_. The intensity maps show: (**a**,**e**,**i**)—G band; (**b**,**f**,**j**)—2D band; (**c**,**g**,**k**)—the ratio I_G_/I_2D_; (**d**,**h**,**l**)—the ratio I_2D_/I_G_ respectively for graphene grown on 4H-SiC (000-1)—(S_1_), 6H-SiC(0001)—(S_3_) and 3C–SiC (100)/Si (100)—(S_2_) substrates. The map columns correspond to S_1_, S_3_ and S_2_ going from left to right. The maps cover a 0.5 × 0.5 mm^2^ area of the sample. The mapping step size was 0.01 mm across x- and y- directions. The rows show the maps of G mode, 2D mode, the intensity ratio determining layers number (I_G_/I_2D_) and the intensity ratio associated with the doping (I_2D_/I_G_) going from top to bottom. The color legend in all maps indicates high intensity in red and low intensity in blue.

In order to address the range in graphene flake quality, the degree of layer homogeneity and layer number, we have employed a surface scanning technique—Raman spectra mapping. Raman spectra mapping can help identify regions with multiple layers, doping, the density of charge and strain-stress effects. Raman mapping collects Raman spectra at specific locations over an area of a sample and allows to correlate the obtained spectra with an optical image of that area. In our specific case, we have imaged and scanned different areas between (100 μm^2^ to 500 μm^2^) of each sample with a mapping step size of 1 μm across the x- and y- directions. We have used an auto focusing adjustment of the laser beam at each point during the mapping acquisition. The results of the Raman mapping studies are combined in Figure 8. The map columns in Figure 8 translate to the three epitaxial graphene samples—S_1_, S_2_ and S_3_—going from left into the right. Top row of Figure 8 (coming from the top)—Figure 8a,e,i illustrates the intensity mappings of the G mode. The second row of Figure 8 (from the top)—Figure 8b,f,j depicts the mappings of the 2D mode Raman shift position. The third row in Figure 8 (from the top)—Figure 8c,g,k shows the maps of the intensity ratio I_G_/I_2D_. The last row of Figure 8 (from the top)—Figure 8d,h,l shows the maps of the intensity ratio I_2D_/I_G_. The color legend in all maps indicates high intensity in red and low intensity in blue.

The G band maps (Figure 8a,e,i) show relatively uniform behavior across the three samples compared to the other maps. This confirms the low sensitivity of the G band to changes in electrical parameters such as doping concentration and charge density. The maps of intensity ratio I_G_/I_2D_ (Figure 8c,g,k) demonstrate the presence of more than a bilayer of graphene in all studied samples (I_G_/I_2D_ >1 or n > 2). All the chosen samples display the same range of layer numbers associated with various flakes dimension and distribution across the surface, which is important for a comparative study. The maps of intensity ratio I_2D_/I_G_ (Figure 8d,h,l) show significant changes across the samples indicating a particular density of charge. The observed density of charge is related to an intrinsic charge redistribution that occurs across the graphene-substrate interface in epitaxial graphene. Particularly, the maps for I_2D_/I_G_ and the 2D band demonstrate a similar behavior in graphene grown on the Si face terminated 6H-SiC (Figure 8f,h) and the cubic polytype 3C-SiC (100)/Si (100) (Figure 8j,l) substrates. For graphene grown on 6H-SiC (0001) this could be attributed to a high degree of growth order imposed by its termination that is, face Si. As described previously, epitaxial graphene grown on 6H-SiC (0001) has a buffer layer which restricts the orientation of the graphene layer along the (0001) axis similar to a Bernal stacking. The high degree of growth order results in a comparable variation in the map intensities of the I_2D_/I_G_ and 2D band across the sample surface. For both samples a reliable distribution of the density of charge is obtained. Due to the absence of the buffer layer and C-face termination of the substrate, we do not expect the same degree of consistency for graphene samples grown on 4H-SiC (000-1) and 3C-SiC (100)/Si (100) substrates. The fact that the behavior of the I_2D_/I_G_ and 2D band across the sample surface in the 3C-SiC (100) system demonstrates an analogous pattern to the one found in 6H-SiC (0001) system is surprising. The observations could be explained by the overall improvement in the growth processes on 3C-SiC (100)/Si (100) substrates, which produces better graphene layer quality and continuity, as reported in the literature [116]. These new, more advanced, growth methods focus on finer control of the graphitization temperature and duration and produce a graphene flake distribution which is similar to the one obtained on the 6H-SiC (0001) substrates. As a result, larger graphene islands could be grown on 3C-SiC (100) substrates than ever before. The I_G_/I_2D_ intensity maps (Figure 8c,k,g) confirm the presence of large homogenous flakes.

The greatest number of differences between the maps are observed for sample S_2_. In particular, there is a disparity between the intensity ratio I_2D_/I_G_ (Figure 8h) and the 2D band (Figure 8f) mappings for S_2_. This can be explained by growth disorder associated with the C-face termination of the substrate. Here, the data illustrate the presence of smaller graphene flakes with various density of charge, although the number of layers is the same as for S_1_ and S_3_. The observed range of the density of charge may be attributed to cluster agglomeration on the graphene surface. Notably, graphene samples grown on 4H-SiC (000-1) showed an increased doping concentration, confirmed by an enhancement of the I_2D_/I_G_ ratio, compared to samples grown on other substrates. This polarity effect is due to the substrate and leads to an increased carrier concentration for graphene grown on C-face (n~1.1 × 10 ^12^ cm^−2^) termination relative to that grown on Si-face termination (n~10^12^ cm^−2^). Here, the layers nearest to the graphene-substrate interface are more strongly doped and dominate the transport properties. The observed behavior of this system is virtually decoupled from the substrate plane. Our results corroborate that epitaxial graphene grown on C-face termination demonstrates superior electrical characteristics compared to graphene grown on Si-face terminated substrate. The conclusion holds even when the number of graphene layers is different.

#### 4.2.1. Electrical Properties of Single Layer Graphene: Capacitor Effect and Gap Opening

The main difficulty in determining the electrical properties of epitaxial graphene arises due to the size and homogeneity of the grown layers. Despite progresses in the growth methods, it is still challenging to grow epitaxial graphene containing large areas with the same number of layers. Usually, graphene layers are randomly distributed in flakes of different sizes and shapes. This becomes clearly apparent when conducting a Raman spectroscopy mapping across an area of the sample surface (see, Figure 8). However, the new shapes of epitaxial graphene also promote novel, emergent electronic properties. For example, Amira and al. reported that epitaxial graphene can exist as islands, domes and bubbles, with unique electronic characteristics [107]. Bubble shapes have also been observed in exfoliated and CVD graphene [124,125], where they have been mainly connected with changes in mechanical properties. Therefore, by controlling the shape of graphene through growth or other methods can give rise to many unusual phenomena with multiple origins—from mechanical to electrical.

**Figure 9:**
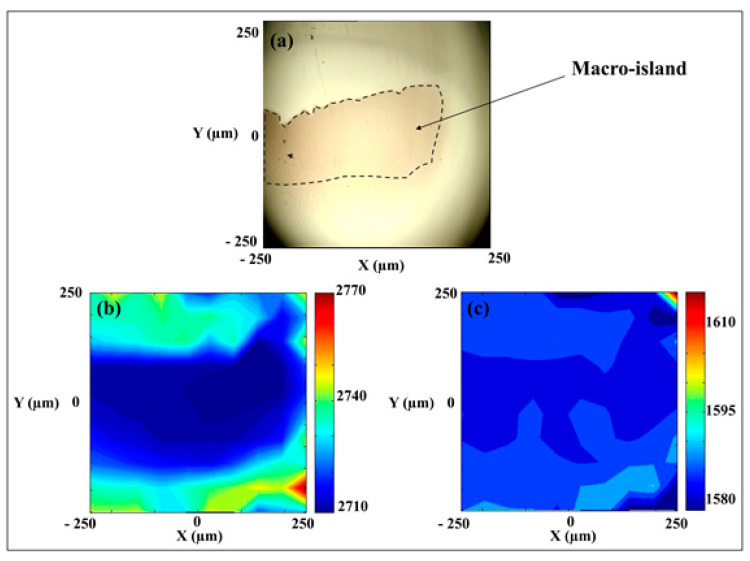
Raman spectroscopy mapping of an epitaxial graphene macro-island, containing four layers: (**a**)—The local optical image. Its size is approximately 400 × 200 µm^2^; (**b**)—the 2D mode position. The 2D shift frequency across the graphene island is substantial ~20–30 cm^−1^ (to lower energies) and cannot be assigned to substrate effect, nor to the variation in the layer numbers; and (**c**)—the G mode Raman shift position. G mode shifts by about 5–7 cm^−1^ to higher energies. The effect can be explained by mechanical changes (strain) in the layer [107].

Raman spectroscopy is extensively used for investigating the effects of graphene shape and size, especially focusing on the variations in the G and 2D bands. As an example, a four layers macro island grown on C face 4H-SiC was investigated using the Raman mapping approach (see, Figure 9) [124]. The island is approximately 400 × 200 μm^2^ in size, (see Figure 9a). The study observed the Raman shift—or changes in the positions of the 2D and the G bands (see, Figure 9b,c) and linked them to variations in mechanical characteristics. The investigation of the 2D band shows a significant shift to lower energies (blue shift) inside the four layers across the macro island (Figure 9b). The 2D band shifts by approximately 20–30 cm^−1^ and cannot be explained by a charge transfer effect from the substrate. A possible reason for the observed shift may be due to a decrease in the electron concentration density as the number of graphene layers is increased. For example, previous works have shown that the Raman shift position of the G mode in single graphene layer decreased by about 7 cm^−1^ (shifts to lower energies) with the addition of another layer [115,126,127,128,129,130]. This occurs due to a depletion of electron density in the graphene sheets. However, in the present study, the opposite behavior is observed for the G band. The G band shifts to higher energies and the band position is increased by about 5–7 cm^−1^ across the island surface. Both 2D and the G bands behaviors indicate that the observed changes in the properties have a mechanical rather than an electrical origin—possibly related to strain. Thus, for graphene and graphene containing structures it is of the upmost importance to correlate the Raman modes analysis with optical and microscopic images of the systems.

**Figure 10:**
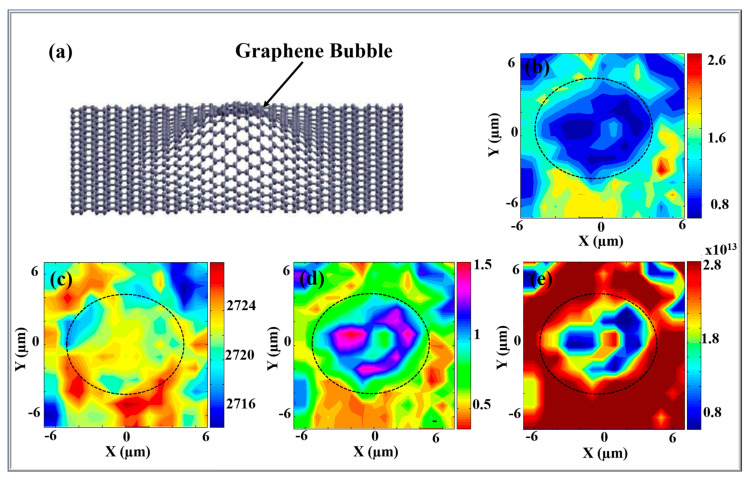
(**a**)—Schematic image of a graphene bubble [134]. Line mapping trajectory is indicated. The Raman mapping of: (**b**)—the intensity ratio I_G_/I_2D_, (**c**)—the Raman shift position of the 2D band, (**d**)—the intensity ratio of I_2D_/I_G_ and (**e**)—the density of charge. The external perimeter of the bubble is shown in a dashed line in (**b**,**e**) [16].

One possible way to strain graphene is to create buckled structures—such as bubbles. Epitaxial graphene bubble—substrate system displays unique electrical properties, that is, the density of charge. Indeed, different density of charges are obtained depending on the charge redistribution between the epitaxial graphene layer and the substrate. The effect arises due to n-type doping in graphene and can influence even substrate layers far removed from the graphene—substrate interface. These properties have been demonstrated using Raman spectroscopy studies in epitaxial graphene bubbles [16]. Amira et al. have investigated the variations of electronic characteristics across a graphene bubble using the Raman mapping technique [16]. The results showing the behaviors of the 2D band at frequency ω_2D_, the intensity ratios I_G_/I_2D_ and I_G_/I_2D_ as well as the associated density of charge across the bubble are summarized in Figure 10. Figure 10a is a schematic image of a graphene bubble with a diameter of ~5 μm—the Raman mapping is conducted in a 13 × 13 μm^2^ area as shown. The epitaxial graphene bubble was produced on 4H-SiC with C face termination. Raman analysis was done using maps on a larger surface area to confirm the existence of the new charge redistribution inside and outside the bubble. Figure 10b shows the intensity (I_G_/I_2D_) across the sample surface and is indicative of the graphene layer number [121]. The intensity ratio of I_G_/I_2D_ shows a clear circular shape roughly corresponding to an area 5 μm × 4 μm which is the bubble. The bubble has a very homogeneous surface and is surrounded by other flakes. Bubble exterior is shown by a dashed line. The low values of the layer number ratio I_G_/I_2D_ < 0.8 indicate that the bubble is formed from single layer graphene. The Raman shift position of the 2D band was examined to identify the local properties of the bubble and to establish the presence of any unique mechanical or electrical characteristics (see, Figure 10c). The shifts in the position of the 2D band are associated mainly with changes in the electrical properties [16] (see, Figure 10c). Figure 10c confirms that the electronic properties inside the graphene bubble are different to those outside its exterior (blue/yellow versus red regions). In order to determine the extent of these differences it is informative to consider the doping ratio I_2D/_I_G_ analysis (see, Figure 10d). Indeed, Geim et al. introduced a method for correlating the intensity ratio of the 2D and G bands I_2D/_I_G_ in exfoliated graphene with density of charge, where the electron and hole doping varies gradually [125]. The examined variations in the I_2D/_I_G_ across the bubble surface and the associated density of charge as described in Reference [26] for single layer graphene are shown in, Figure 10d,e. The results show a consistent density of charge across the bubble. The intensity ratio I_2D/_I_G_ has weak intensity on the top and the edges of the bubble, while the intensity is higher everywhere else (compare green and pink areas in Figure 10d). The I_2D_/I_G_ intensity ratio ranges from 0.5 to 1.5 (see, Figure 10d) [16]. By using the Geim et al. method to correlate the I_2D_/I_G_ with the charge density, it was established that the electron density varies between 0.75 × 10^13^ cm^−2^ and 3 × 10^13^ cm^−2^ and that the electron density is higher at the center of the bubble (see, Figure 10e). These results prove that in the formation of the bubble the graphene layer becomes unintentionally doped. The effect may be attributed to an emergent graphene-substrate interaction at the interface that gets amplified due to strain as the graphene layer becomes buckled and deformed.

The physical origin for the observed phenomena may lie in a new phonon–plasmon coupling where the charged graphene layer–substrate interface acts similarly to a parallel plate capacitor with charge separation. By analogy with a capacitor, the graphene layer–substrate interface also demonstrates an increase in the internal electric field as well as a possible gap opening [123,124,125,126,127,128,129,130,131]. Specifically, in this case, the surface of the SiC substrate plays the role of a highly charged thin layer while the graphene bubble stores charge of the opposite sign. The newly observed density of charge is highly descriptive of such a capacitor behavior. The density of charge illustrates that the graphene layer becomes naturally n-doped (i.e., electron doped), forming one plate of the capacitor, while positive charges accumulate on the surface of the SiC substrate, forming the second opposite capacitor plate. There is no overall charge and the configuration is stable. The density of charge in the bubble confirms overall charge neutrality. The observed charge redistribution could arise due to strong Coulomb coupling at the epitaxial graphene–substrate interface. Interestingly, the charged graphene–substrate interface may behave as a resonance cavity for plasmon excitations, by virtue of mirroring the electromagnetic radiation entering the bubble interior from the outside. Plasmonic excitations may induce possible magnetic field changes inside the bubble. Recently, it has been shown that local strain due to graphene buckling around triangular Pt nano-islands may result in the formation of gigantic, alternating pseudo-magnetic fields [121]. Depending on the shape of the bubble [132,133] and the surrounding flakes similar magnetic effects may be relevant here.

The newly discovered capacitor effect around epitaxial graphene bubble - substrate interfaces may lead to other unconventional electrical properties. For example, it may result in the possible opening of a gap. It is known that free standing graphene does not display any gap or intervalley division at the Dirac cones due to the lattice distortion. This latest must be transverse otherwise the graphene layer keeps its properties. Indeed, the breaking of the symmetry must affect graphene sublattices otherwise graphene layer remains invariant [123]. In Reference [126] it has been shown that in epitaxial graphene a possible gap opening may appear due to the symmetry breaking between two main graphene layer sublattices, triggered by the substrate. Thus, the emergence of gap opening could only be attributed to the existence of a substrate. Epitaxial graphene bubble is considered as free-standing graphene. Here, the graphene–substrate interaction leads to symmetry breaking and the emergence of a gap in the Dirac spectrum [123,126]. Indeed, the substrate plays the role of additional van der Waals forces affecting the transverse lattice displacement of the graphene atoms. This effect may arise even in C face terminated epitaxial graphene, despite the absence of a buffer layer [126,135]. Reference [16] determined the opening gap ∆ based on the electrostatic field inside the bubble. The expression for the gap could be written as:

(1) $$\Delta \;=\frac{2e^{2}a\;n_{Gr\;\;}}{\varepsilon }\;,$$

where the electric field *E* is given by to $E=\frac{e\;n_{Gr\;}}{\varepsilon }$. *ε* corresponds to the dielectric constant of the SiC substrate and *e* is the electron charge, while *a* = 0.5 Å represents the quantum buckling of the graphene layer [126]. Based on this expression a local charge density $n_{Gr\;}$ = 0.75 × 10^13^–3 × 10^13^ cm^−2^ could be identified inside the graphene bubble for a permittivity value of $\;\varepsilon_{r}$ = 10. The corresponding electric field varied between 0.10 and 0.6 × 10^9^ V m^−1^. Using Equation (1), the gap values are found to lie in a range between 1.4–5.4 meV. Thus, the gap is extremely small and its origins may be related to weak local fields present in the system. Previously, in Reference [42] the gap opening in epitaxial graphene grown on Si-terminated face SiC was attributed to the presence of the buffer layer. In the present case of graphene grown on carbon terminated face there is no buffer layer. Indeed, the bubble structures are formed in a freestanding way on the substrate. The emergence of gap opening for graphene grown on carbon face termination could be explained by the presence of impurities or strain. Thus, the existence of graphene bubbles will accelerate the development of nanotechnology based on different density of charges such as transistors [124]. The main findings indicate that Raman spectroscopy and in-depth Raman analysis may be used as a non-invasive method for studying the changes in electrical and structural properties in epitaxial graphene arising due to: deformation, impurities or strain, as evidenced by recent work on epitaxial graphene bubbles [126].

#### 4.2.2. Electrical Properties of Single Layer Graphene: Substrate Investigation

Motivated by the strong electronic correlation that exists at epitaxial graphene—substrate interface, we have shifted the focus of our investigation on the changes in the SiC substrate. Generally, the electrical properties in graphene have been determined through the study of graphene Raman modes similar to Geim and et al. method. The investigation of the substrate Raman modes has always been ignored for all graphene types. In Reference [126], we have reported for the first time the possible imaging of the local graphene density of charge through the longitudinal phonon plasmons coupling (LOOPC) mode in the substrate. Specifically, we have investigated the behavior of the longitudinal optical phonon (LOP) mode of 4H-SiC substrate denoted A_1_ (LO). We have demonstrated that the 4H-SiC LOP-plasmons modes depend extremely on the local coupling that exists between the graphene and the substrate (LOOPC). The suggested LOOPC method is based on correlating the energy shift position of the A_1_ (LO) phonons to the corresponding charge carrier in epitaxial graphene layer. The carrier density is determined using a classical method defined by Geim and et al. detailed above [127]. Indeed, the LOOPC is measured in the frequency range between [100–1000 cm^−1^], where 4H-SiC (000-1) substrate Raman signature exists. The Raman modes shift position of the substrate depend on the used laser. In our study, we have used a 488 nm Ar-ion laser as an excitation source. Different Raman bands of the 4H-SiC substrate have been located, particularly, we have located A_1_ (LO) band at around 967 cm^−1^ [128,129]. It is important to note that the LOOPC method has been widely used to determine the density of charge in SiC materials. However, it has never before been used to investigate the electrical properties in epitaxial graphene. This method describes the density of charge in a charged volume. In the case of epitaxial graphene, the volume refers to the few layer of SiC adjacent to the graphene—substrate interface. We have illustrated that these layers are naturally charged and doped. The contributing layers are localized at about 1 to 2 μm thickness depth in the SiC substrate areas in contact with graphene. Here, the high precision of the layer thickness is assigned to the high-resolution confocal Raman spectrometer used in our measurement. Actually, this Raman spectrometer laser may penetrate to about ⁓1 to 2 µm below the top layer of the substrate. Therefore, the LOOPC method, used to describe electronic properties in graphene, correlates the Raman shift position changes of the A_1_ (LO) band to the local charge carrier concentration [125]. The phenomena arise because, plasmonic waves induce an oscillating charge density in the system that affect A_1_ (LO) phonons and polarize graphene at the same time. Phonon—plasmon coupling could be verified by a simple comparison of the Raman shift in the A_1_ (LO) mode located at 962 cm^−1^ between a freestanding and graphene-coupled 4H-SiC. The LOOPC modes intensity I(ω)  are described using the theoretical method given by the Equation (2):

(2) $$I\left(\omega \right)=A\left(\omega \right)Im\left(-\frac{1}{\varepsilon \left(\omega ,q\right)}\right)\;,$$

where, A(ω)  and ε(ω,q)  are correspondingly the spectral and dielectric functions. Im  represents the imaginary part of $\left(-\frac{1}{\varepsilon \left(\omega ,q\right)}\right).$ These latest depend on the $\omega_{p}\;$ plasma frequency, $\gamma_{p}$, plasmon damping concentration, $\Gamma_{p}$ damping constant of electrons, $\varepsilon_{\infty }$ high-frequency dielectric constant, C Faust–Henry constant and ($\omega_{L})$ of the 4H-SiC substrate modes: TO (LO) and A_1_[LO] (E_1_ [TO]). All the changes of the LOOPC mode are associated with changes in its shift position and its line shape [127,130]. During the theoretical fit of the LO Raman bands we have used parameters such as: the electron effective mass in SiC is m* = 0.29 *m*_0_ (*m*_0_ is the free electron mass) and the frequencies of the TO (LO) phonons,$\;\omega_{T}\;$= 744 cm^−1^ ($\omega_{L}$ = 962 cm^−1^), in an undoped 4H–SiC substrate [136]. 

**Figure 11:**
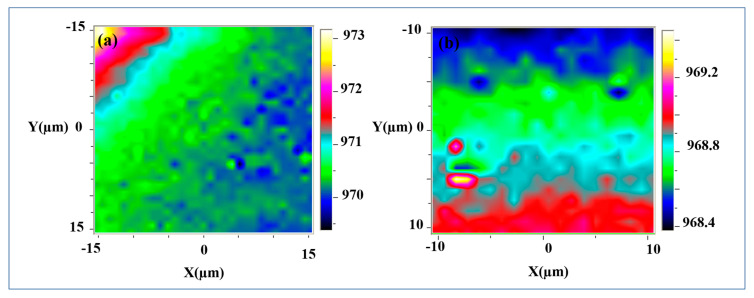
A_1_ (LO) modes Raman shift position in the different areas [126]. (**a**)—single graphene layer A_1_ A_1_ (LO) mode position demonstrates blue-ward shift by approximately 4 cm^−1^; (**b**)—bilayer graphene A_2_—A_1_ (LO) mode position demonstrates blue-ward shift by approximately 1 cm^−1^ Similar dependencies of the A_1_ (LO) mode are observed in graphite A_3_.

The LOOPC method precisely describes the density of charge in the graphene layers. The LOOPC method was successfully employed to describe the electronic properties in graphene with different layer numbers [126]. The work in Reference [126] reports mainly the results for four graphene-related areas—A_1_, A_2_, A_3_ and A_4_. A_1_ and A_4_ correspond to two single graphene layers; A_2_ is bilayer graphene and A_3_ is graphite. Measurements of A_1_, A_2_ and A_3_ belong the same area of the sample whereas A_4_ is obtained from different area (see, Figure 11a,b). The LOOPC coupling is identified through the shape and broadening of A_1_ band as a signature of the density of charge changes. A_1_ (LO) investigation in the different studied areas demonstrates its blue shift and broadening. This occurs due to an increase in the phonon–plasmon interaction. Heating effect is excluded as a possible cause for the observed changes in the A_1_ (LO) mode due to the high control of the growth process. However, in some cases, A_1_ (LO) mode was not properly fitted. For example, for the single layer A_1_, bilayer A_2_ and graphite A_3_ the tail of the A_1_ (LO) mode did not fit well with the model. This is contrary to the observations in the single layer A_4_ where the entire shape of the A_1_ (LO) mode was reproduced correctly by the LOOPC model. The authors suggested a possible Voigt fit to improve the fit of the A_1_ (LO) mode. For the single graphene layer A_1_ the investigation of the Raman shift position of A_1_(LO) illustrates a significant blue shift of almost 4 cm^−1^ at around 971 cm^−1^ compared to that of the undoped 4H-SiC (see, Figure 11a). For the bilayer graphene A_2_, the A_1_ (LO) mode shift position varies by about 1 cm^−1^ in the frequency range 968.4–969.4 cm^−1^ which is almost constant (see, Figure 11b). Similarly, for the graphite A_3_, A_1_ (LO) mode shift position changes by about 1 cm^−1^ and is nearly constant (see, Figure 11b). However, for the single layer A_4_ the A_1_ (LO) mode appears at frequencies much closer to the frequencies in the undoped 4H-SiC substrate [964 cm^−1^ and 967 cm^−1^] and demonstrates much smaller variation compared to A_1_. Note, that the theoretical model fits particularly well for single layer A_4_. of the observed differences in the Raman shift position of the A_1_ (LO) mode are assigned to different density of charge. Thus, we have revealed a highly sensitive theoretical model that may describe the local graphene charge carrier through substrate imaging. The experimental findings confirm the premise that graphene has very unique quantum capacitance that induces new charge distribution in the graphene-substrate interface. Thus, we have extrapolated that the improper fits of the A_1_ (LO) mode in A _1,2_ and A_3_, may be explained by a high local charge density fluctuation, arising from the capacitance effects, while the correct fit of the A_1_ (LO) mode in A_4_ may be due to weak doping in such areas. 

**Figure 12:**
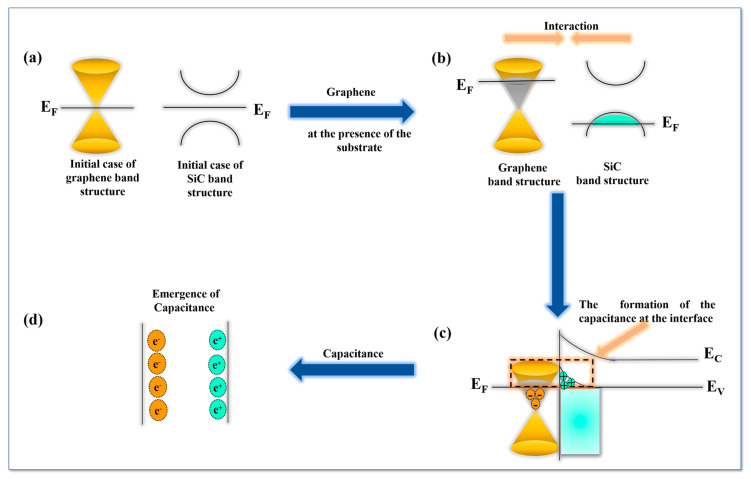
**Capacitor effect in epitaxial graphene:** (**a**)—electronic bands in graphene and SiC substrate separately. The graphene spectrum shows the Dirac point at the Fermi level, while the SiC spectrum shows a gap; (**b**)—graphene -SiC system interactions induces changes in the bands. The Dirac cone positioned under the Fermi level, corresponding to n-doping in graphene. The SiC conduction band moves above the Fermi level, corresponding to hole doping; (**c**)—The charge redistribution at the graphene-SiC interface results in negatively charged graphene and positively charged SiC surface layer; (**d**)—The density of charge leads to the emergence of the capacitor effects at the graphene-substrate interface, which can be modelled as a parallel plate capacitor.

The analysis of the A_1_ (LO) mode broadening is at the limit of the LOOPC model and will not be discussed here. In any case, the mode broadening behavior does not affect the description of the density of charge presented here. Our evaluation of the LOOPC method has established that in weakly doped regions the A_1_ (LO) mode position shift follows generally the shift of the graphene Raman modes. However, in certain regions, we have observed a particularly large difference between the substrates A_1_ (LO) mode and the graphene’s G band. This has been attributed to a large electron density variation in the corresponding areas. The proposed large charge density fluctuations may be induced by the strong screening effects from the Coulomb forces between the substrate and graphene. Thus, the LOOPC technique is an advanced optical method based on Raman spectroscopy analysis giving evidence for the phonon–plasmon coupling in epitaxial graphene. This simple method allows us to image properly the density of charge at the epitaxial graphene–SiC interface system, based purely on the behavior of the substrate Raman modes. In addition, it can be used to determine the free carrier concentration *n* in the graphene layer. This can be done by investigating the 4H-SiC plasma frequency $\omega_{p}$ that could be written as [137]:

(3) $$\omega_{p}^{2}=\frac{4\pi ne^{2}}{m^{*}\varepsilon_{0}\varepsilon_{\infty \;}}+\frac{3}{5}{\left(q\vartheta_{F}\right)}^{2}.$$

where, m* corresponds to the electron effective mass in SiC (m* = 0.29 *m*_0_, *m*_0_ is the free electron mass),$\;\varepsilon_{0\;},$ the dielectric constant of the vacuum,$\;\varepsilon_{\infty \;},\;\mathrm{the}\;\mathrm{high}-\mathrm{frequency}\;\mathrm{dielectric}\;\mathrm{constant}$, *q*, the crystal momentum and $\vartheta_{F}\;\mathrm{is}\;\mathrm{the}\;\mathrm{Fermi}\;\mathrm{velocity}$. The electron concentration *n* can be determined by adjusting parameters $\omega_{p}\;and\;\gamma_{p}.\;$ The plasma frequency $\omega_{p}$ and the plasmons damping $\gamma_{p}$ for graphene areas A_1_, A_2_, A_3_ and A_4_ were obtained previously [126]. Using the described approach, the charge density in the different graphene areas corresponding to A_1_, A_2_, A_3_ and A_4_ was found to be *n*_1_ = 2.7129 × 10^18^ cm^−3^, *n*_2_ = 2.5214 × 10^18^ cm^−3^, *n*_3_ = 4.1 × 10^18^ cm^−3^ and *n*_4_ *=* 1.8857 × 10^18^ cm^−3^, respectively. The charge density for the undoped 4H-SiC substrate was calculated as *n_substrate_* = 3.448 × 10^11^ cm^−3^. The obtained results were compared to the charge density values deduced from the graphene G band position shifts and showed excellent agreement with previous findings [126]. The LOOPC method has additional advantages over the conventional graphene Raman modes investigation and can present information about the precise thickness of the substrate contributing to the graphene-substrate interface. This is not possible using the standard graphene Raman spectroscopy approach. Based on the electroneutrality arguments for the graphene—substrate system, we can determine the thickness of the charged substrate layer ’L.’ The graphene layer is doped with electrons and is, therefore, negatively charged. For neutrality, the SiC substrate must be positively charged. This gives a simple relation for the charge densities in the graphene and the participating substrate layer *n_gr_* = n_substrate_* L. Using this expression, we find that the thickness of the charged surface layer of the SiC substrate is approximately L = 0.54 mm which is equal to 2857 SiC bilayers. All these results add further proof to the capacitor effect emerging at graphene-substrate system (see, Figure 12). Note, that the plasmonic excitations in graphene near a Dirac point for small plasmonic momenta q could be written as [71]:

(4) $$\omega_{pgr}^{2}=\frac{2\;n_{gr}e^{2}}{m_{gr}^{*}}q+\frac{3}{4}{\left(q\vartheta_{F}\right)}^{2},$$

where $m_{gr}^{*}$ is the electron effective mass at the bottom of the graphene band and *n_gr_* is the local charge carrier concentration in the graphene layer. We neglect the possible hybridization effects, which is outside of the focus of the present study. 

Actually, graphene layers–charged substrate layers system behaves as a resonant cavity for plasmon excitations. These plasmon waves move along the graphene surface. Previously, we have discussed the emergence of capacitor effects in an epitaxial graphene bubble. However, similar phenomena may be observed at the graphene-substrate interface due to other contributing electrostatic factors, such as substrate defects, charged impurities and charged puddles of epitaxial graphene that capture charges due to the presence of SiC step terraces [138,139,140]. These factors induce a permanent gate voltage that affects the different charges in the vicinity of the graphene layer. The effect of the gate voltage and its relation to the band structure and capacitance properties at the graphene-SiC interface is summarized in Figure 12. 

For epitaxial graphene bubble, the appearance of local electrostatic field leads to a gap opening and capacitor formation. The capacitance of the whole system may be separated into the sum of the capacitances of each component. C-face terminated epitaxial graphene interface system is composed of graphene layers and doped SiC layers. Therefore, the total capacitor could be written as the summation of a quantum capacitor $C_{Q}$ and an electrostatic capacitance $C_{eq\;4H}$ associated respectively with the graphene layer and the doped layers of 4H-SiC substrate. The quantum capacitor $C_{Q}$ depends only on the charge density of the graphene layers, which is related to its density of states DoS [16]. The capacitance of the charged SiC layer $C_{eq\;4H}$ can be treated as a conventional electrostatic parallel plate capacitor, which depends on the layer charge and inversely on the separation between the layers. The charged SiC layer properties differ based on the face termination of the graphene growth. The C-face terminated graphene has no buffer-layer contrary to the Si-face terminated graphene. The electrostatic effects of the buffer layer must be included in the overall calculation where appropriate. Thus, we must add an extra electrostatic capacitor $C_{eq\;B}$ associated with the buffer layer to describe the Si-face terminated graphene system. However, for C-face terminated graphene no buffer term is necessary [139], so the buffer capacitance contribution can be set to zero ($1/C_{eq\;B}=0).\;$ The capacitance at the graphene-substrate interface can be modelled as a combination of the capacitances of its isolated components added in series. Consequently, the systems total capacitance *C* becomes [141]:

(5) $$\frac{1}{C}=\frac{1}{C_{Q}}+\frac{1}{C_{eq\;\;4H}}.$$

Here, the quantum capacitance $\;C_{Q}$ depends on the density of states at the Fermi energy $E_{F}$. The Fermi energy $E_{F}$ can be calculated using the Fermi velocity $\upsilon_{F}\;\mathrm{of}\;\mathrm{about}\;$10^6^ ms^−1^ in graphene. Thus, we found that $C_{Q}$ could be defined as [142]:

(6) $$C_{Q}=\frac{2Ae^{2}\left|E_{F}\right|}{\pi \hbar^{2}\upsilon_{F}^{2}},$$

where, *A* is the area of the plates of the capacitor and ℏ corresponds to the Planck constant (ℏ = 6.581016 eV s).

The chemical potential μ at zero applied electric field is equal to the Fermi energy at low temperatures, where both depend on the graphene layer charge. This dependency could be written as below [142]:

(7) $$\mu =E_{F}=\hbar \;\vartheta_{F}\sqrt{\frac{\pi n}{2}},\;\mathrm{where}\;\mathrm{the}\;\mathrm{electron}\;\mathrm{density}\;\mathrm{is}\;n=\frac{g\;E_{F}^{2}}{4\pi \hbar^{2}\upsilon_{F}^{2}}.$$

Consequently, we may also determine the Fermi energy and the associated quantum capacitance per unit area. From Reference [126], we have reported that the *E_F_* = 0.14 eV and the $\frac{C_{Q}}{A}$ = 1.71 × 10^−4^ mF m^2^ for the single layer graphene in A_1_. Due to its small value the quantum capacitance dominates the total capacitance at the interface [23]. This effect is known as graphene quantum capacitance effect and it was previously observed in different types of graphene [143]. We may also determine the electrostatic capacitance of the SiC substrate given by:

(8) $$C_{eq\;4H}=\frac{\varepsilon A}{\ell_{i}}\;,$$

where ε is the substrate permittivity, *A* represent the area of the substrate surface and $\ell_{i}$ associated with the bond size in SiC (i.e., Si-C _bond size_ ≈ 1.89 Å). We have found that the electrostatic capacitance of SiC per unit area is equals to $\frac{C_{eq\;4H\;}}{A}$ = 1.64 × 10^4^ mF m^2^ [126]. The $C_{eq\;4H}$ is several orders of magnitude greater than the quantum capacitance *C_Q_* confirming our conclusions that the graphene effects dominate at the interface. Therefore, the contribution of $C_{eq\;4H}$ will be negligible in the total capacitance. This explains the vital role that quantum capacitance effects play in the total electrical properties of epitaxial graphene [144]. It important to highlight the existence of mini gap opening here, which has been previously discussed for the epitaxial graphene bubble and is schematically depicted in Figure 12 [126].

## 5. Future Challenges for Raman Spectroscopy to Study Effects of the Lattice Strain or Pseudo-Magnetic Field on Relativistic Dirac Fermions

### 5.1. Quantum Hall Effect (QHE) and Raman Spectroscopy

One of the most amazing properties of graphene is Quantum Hall Effect (QHE), which is usually observed in magneto-transport measurements at low temperatures [145]. Such studies have shown that most flat graphene films grown by proton-assisted chemical vapor deposition (CVD) may exhibit QHE even at room temperatures [145]. The flatness of the CVD graphene was a crucial component in this discovery. Usually, QHE is intimately related to the Landau quantization arising in graphene when the transverse magnetic field is applied. The Landau Energy levels spectrum has a very simple form,$\;E_{n}\sim \pm v_{F}{\left(2ehB\left|n\right|\right)}^{\frac{1}{2}}$, where *n* is an arbitrary integer. Raman spectroscopy provides a unique tool to detect the Landau quantization, determine the Fermi energy and reveal electron correlations which may influence the Landau quantization. Thus, in-depth analysis of the Raman spectra may establish the effective distance between the Landau levels and reconstruct the entire Landau spectrum. Indeed, such unique experiments have been done by Potemskii et al. [26] and reveal some nontrivial behavior of the Fermi velocity. It was suggested that the deviation from the fundamental Landau Quantization Law in graphene, ($E_{n}\sim \pm {\left|n\right|}^{\frac{1}{2}}$), was probably related to the electron-electron correlations [26].

### 5.2. Graphene Flatness, Nano Domes and Associated Lattice Strain- Geometry Dependent Pseudomagnetic Field and Quantum Spin Hall Effect (QSHE)

However, the graphene as any two-dimensional (2D) crystal cannot exist in a flat state unless an external flattening support is applied. One example to preserve the flatness of graphene is to sandwich (or confine) it between two BN layers. According to the Mermin-Wagner theorem, many unconfined 2D material may experience all sorts of 3D distortions leading to the formation of nanodomes, wrinkles, nano-valleys (see the discussion in Reference [123]. When graphene surface is not flat and takes on some shape another series of analogous phenomena can arise even without external magnetic field. Note that the shape of the graphene surface plays a key role in its properties and is always associated with the creation of lattice distortion. Such lattice strain is equivalent to a vector potential in the Dirac equation describing the electron states. Depending on the lattice strain different electronic states may arise. In particular, specific types of strain may act in the same way as a transverse magnetic field [120,146]. In this case, of course, a system of discrete energy levels will appear, similar to those arising at Landau quantization. Consequently, the energy spectrum will follow the same simple quantization law $E_{n}\sim {\left(\left|n\right|\right)}^{\frac{1}{2}}$. This effect has been observed extensively in graphene [120,146] and in phosphorene [147]. 

Interestingly, in this particular case, there is no breaking of the time reversal symmetry, which always happens in magnetic field. Effectively, this means that the electrons with opposite spins are always moving in the opposite directions. As a result, a unique situation arises where the spin-up and spin down electrons have opposite Chern number. The locking of the edge states follows and generates spin current only, with no accompanying charge transfer. The behavior describes a Quantum Spin Hall Effect (QSHE). The phenomenon was first theoretically predicted by Kane and Mele in 2007 [148] and then later experimentally observed [149].

The nano-dome or buckled deformation in graphene leads to localized lattice strain in regions surrounding the distortion. Such strain mimics effectively a large inhomogeneous magnetic field. Note, that there no actual magnetic field present and this phenomenon is called a pseudo-magnetic field. By controlling the shape of the graphene deformation, it is therefore possible to adjust the strain and the homogeneity of the magnetic field. The simplest way to achieve this is by depositing nanostructures of an appropriate form on the substrate underneath the graphene layer—creating a landscape and removing its flatness. Recently it was shown that when graphene is placed on such a textured substrate it is possible to manipulate the formation of topological states, such as edge and snake states [150]. The method of nanoscale strain engineering [151] opens a new era in the creation of various devices, based on topological phenomena which can operate even at room temperature. The epitaxial graphene on SiC has a naturally occurring nano-domes structure and associated lattice strain, which depends on the method of its growth [16]. Thus, in epitaxial graphene the nanoscale strain engineering can be performed just by adjusting its growth process.

### 5.3. SiC Termination Impact on Landau Quantisation Localised by Raman

Raman spectroscopy is powerful non-destructive characterization technique and may properly address localized electron states in epitaxial graphene with nano-domes, where lattice strain is mimicking a huge, inhomogeneous pseudo-magnetic field. Such states localize electrons, similar to Landau levels and are of various intrinsic origins, related to lattice strain. We expect that around graphene nano-domes, due to the presence of the strong pseudo-magnetic field, there may exist both edge and the snake states as well as other topological defects still waiting to be discovered. Similar unique behavior has been already been observed in a real transverse magnetic field.

The existing Raman experiments [152] also demonstrate that Landau quantization depends on the SiC substrate termination. This occurs because the covalent bonding between Si and C atoms is strongly polarized. Therefore, the Si-C bond may be viewed as the equivalent of a local dipole. As a result, neighboring Si and C layers represent a series of polarized dipoles. The orientation of the dipoles depends on the termination of the SiC substrate. For example, the positive end of the dipole is facing the surface for Si termination and is directed towards the bulk for C termination. The ferroelectric orientation of these dipoles is obviously related to the formation of a local polarization or an intrinsic electric field. This electric field is acting on the graphene epitaxied on the surface of the SiC substrate and may create a gap in the electron spectrum. Thus, depending on the type of the substrate termination the graphene doping, induced by the local electric field, may have either an electron or a hole character. For ideal graphene there is no difference between electron and hole doping. While for graphene grown on a substrate the presence of impurities, like donors and acceptors, can create different scattering properties for electron and hole carriers. Therefore, it is expected that the electrical properties in epitaxial graphene will be strongly affected by the SiC substrate termination. This is exactly what is observed.

### 5.4. Landau-Phonons Level Crossings

Furthermore, the investigation of the Landau levels in graphene has been done with the use of Raman spectroscopy, by following the behavior of the E_2g_ and the G bands. Indeed, under a magnetic field the G-band displays a mode doublet where one Raman mode remains fixed and the other mode oscillates depending on the value of the applied field [26]. It has been shown that the discovered oscillatory component of the G-band is responsible for the anticipated magneto-phonon multi resonance [153,154]. The change in the frequency and width of this band can be understood in terms of a series of energy level crossings. It happens each time when the G-phonon energy matches the energy of a specific transition between Landau levels or, in other words, inter-Landau level excitations occur [155,156]. The Landau-phonon level crossings are defined by appropriate selection rules derived from the symmetry or associated irreducible representations [157].

The analysis of the Raman results allows to develop a better understanding of the electron-phonon interaction in the investigated graphene system [158] as well as novel quantum phenomena, such as fractional quantum Hall effect [159]. Observation of the fractional quantum Hall effect in graphene due to strain is still waiting to be discovered. Important to note that in the strained system it is possible to observe the fractional quantum spin Hall effect since the strain does not break time reversal symmetry. Graphene is a unique material where the effects of a magnetic field of any strength can be induced by lattice strain. The proper strain engineering in graphene opens new horizons in physics, where the influence of gigantic magnetic fields—surpassing by orders of magnitude all existing technologies and never previously observed in nature, can be studied in detail.

It is well known that two-dimensional electron gases, similar to graphene, when exposed to a magnetic field may absorb electromagnetic radiation via electronic transitions between Landau levels [160]. Recently, it was shown magneto-transmission and Faraday rotation in high-mobility encapsulated monolayer graphene is strongly enhanced both in the infrared and terahertz frequency ranges. In particular, it was demonstrated that nearly 50% of the light absorption is observed in encapsulated graphene, compared to only 2% in pure graphene. Moreover, in this system, the magnetic circular dichroism is about 100% and Faraday rotation is record high [160]. By analogy, the presence of a pseudo-magnetic fields, due to strain, may also enhance the light absorption, the magnetic circular dichroism and Faraday rotation. Indeed, recently it was found that epitaxial graphene with nano-domes displays strong photo-response [161]. A possible origin of the observed behavior may be due to lattice strains created by nano-domes in graphene.

### 5.5. Homogeneous Strain Originated by Kirigami Art, Perforated Graphene and Epitaxial Graphene-SiC Intercalation: Future Raman Challenges

Other forms of the lattice strain exist in graphene and graphene-related materials, such as: 1-epitaxial triangular nano-prisms [152] and 2-nano holes induced in CVD graphene bilayers with plasma etching [162] 3-epitaxial graphene-SiC intercalation [163]. It was also shown theoretically that reshaping graphene layer with the kirigami (cut-and-fold Japanese art) method provides another source of lattice strain due to a redistribution of the infinite gaussian curvature from conical points [15]. Indeed, such a complex landscape of the graphene surface is accompanied by very nontrivial and complicated lattice deformations [144]. This type of buckled surface landscape is formed by growing epitaxial graphene on a 6H-SiC substrate covered by a dense array of triangular nano-prisms that create local lattice distortions [152]. The nano-prisms appear during the epitaxial growth process. Their shape and distribution are controllable by the Argon flow in the chamber [162]. The resulting deformed monolayer graphene contains regions of high strain in the vicinity of the nano-prisms. It was found that tiny triangular nano-prisms on the surface of the SiC may induce strain which can mimic uniform magnetic fields of about ~41 T [152]. Such large field enhanced Landau quantization, where the largest separation occurs between the zero and the first Landau levels. The level separation is so large that it can be detected by Angle-Resolved Photoemission Spectroscopy (ARPES) even at room temperature. The buckled graphene contains areas with strong strain or a pseudo-magnetic field and those without strain, as in conventional graphene. That is clearly evidenced by the ARPES spectrum observed from the spot-size of about 1 mm^2^, which shows a combined spatial average over strained and unstrained regions of graphene. In fact, several distinct Landau level spacings were observed, which follow the Landau quantization Law for graphene’s massless Dirac electrons. The strength of the pseudo-field, extracted from the strain, was about ~41 T [120], which is extremely difficult to obtain with a real magnetic field. The observed results are very general and indicate that the phenomenon of graphene deformation or buckling into nano-domes is always associated with lattice strain and the creation of the large pseudo-magnetic field regions. The field orientation in different regions changes sign and averages out to zero, in general.

**Figure 13:**
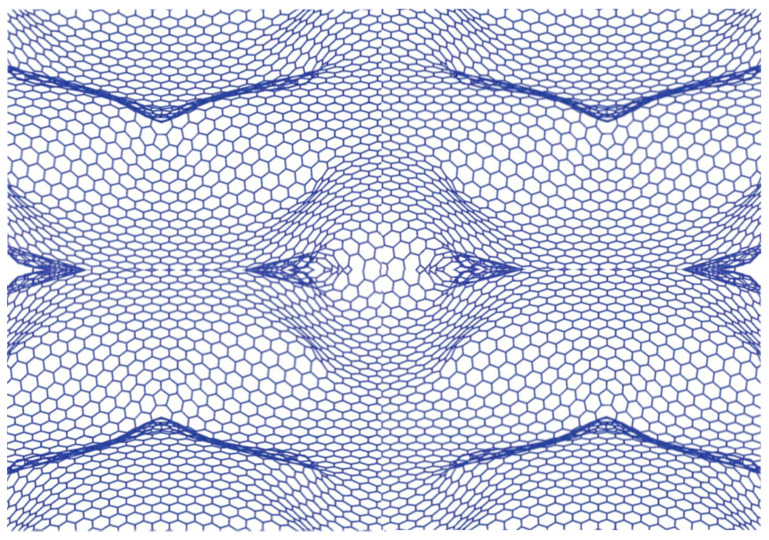
Illustration of graphene buckling landscape obtained through deformation strain and/or perforation. The image reproduced from [*Phys. Today* **2020**, 73, 46–52], with the permission of the American Institute of Physics. doi.org/10.1063/PT.3.4569.

Similar phenomena have been reported in bilayer CVD graphene perforated by Nitrogen (N_2_) plasma [162]. Here, the bilayer has a quasi-free-standing nature, that is, suspended above a substrate, as demonstrated by Raman analysis. However, N_2_ plasma etching of the CVD graphene can produce surface structures similar to bubbles and nano-prism deformations in epitaxial graphene. During this plasma treatment many holes appear in the top layer, where the edges of the holes glue to the bottom of the basal plane of the graphene layer. Such edges become comparable to the foundations found in bubbles of epitaxial graphene. Similar phenomenon has been located in newly designed quasi free-standing epitaxial graphene layers. This type of graphene has been developed through epitaxial Graphene-SiC intercalation. Such a challenging method is based on the decoupling of the graphene layer from the buffer layer-SiC interface via several intercalants [163]. These latest plays main role in the detachment of the graphene layers from the zero layer or the so-called buffer layer. Diverse classes of intercalants have been used, mainly, H- and O-intercalation have been commonly probed [164,165,166,167,168]. Here, the obtained epitaxial graphene become close to the discussed CVD graphene changed through N_2_ plasma etching. Indeed, the free-standing nature of the grown graphene layers obtained by both techniques makes them comparable to reshaped graphene layer, that is, bubbles and pyramid, where similar properties exist. More directly the plasma etching perforation and epitaxial graphene-SiC intercalation may be related to theoretical predictions of graphene Kirigami [15]. (Kirigami is a traditional Japanese paper art [15] that includes folding, cutting and gluing.) The designed Kirigami structures are based on cutting a graphene sheet and forming numerous graphene edges that are glued together healing the damaged segments. This folding around the cuts creates conical shapes which usually have an infinite Gaussian curvature at the conical point. However, due to the graphene lattice deformations, this singularity in Gaussian curvature becomes distributed in a region around the conical points, leading to the formation of a very complex graphene landscape (see, Figure 13). These new graphene shapes demonstrate high internal strains which can mimic the effect of gigantic pseudo-magnetic fields >300 T [15,120]. Here, strain results in pseudo-magnetic fields of different sign depending on the deformation and the local position with respect to the graphene hexagons.

A homogeneous, point-like, local deformations result in a pseudo-magnetic field which changes sign according to the C_3v_–symmetry transformation around the point. The axis of the symmetry is perpendicular to the graphene surface [120]. Conversely, to create a homogeneous pseudo-magnetic field a lattice deformation of the special C_3v_–symmetry is needed [120]. By introducing this type of deformation, it may be possible to stabilize a graphene p-n junction that will behave as if a uniform transverse magnetic field is present [120]. In this manner, simply by utilizing strain, multiple p-n junctions with topological edge and snake states could be established in the complete absence of a real, external magnetic field [150].

For arbitrary graphene lattice deformations, the pseudo-magnetic fields will exhibit a spatial variation, which is randomly changing sign and averaging to zero. In such situations, according to the Aharonov-Casher-Dubrovin-Novikov theorem [169], a special form of Landau quantization will arise, establishing a zero energy Landau level. The origin of this zero level is in the interplay between spin and orbital degrees of freedom. The non-zero Landau energy levels will be arbitrary and they will be separated from the zero Landau energy level by a reasonable gap associated with the amplitude of the spatial variation of the pseudo-magnetic field. In other words, the creation of the zero energy level will be robust [169]. As a result, the zero energy Landau level may trap Majorana fermions (MF) [170,171,172,173]. These elusive particles are also their own antiparticles and were predicted by Ettore Majorana [173] and can only occupy a state with zero energy. The zero-energy Landau level satisfies this condition, exactly. The search for MF is rapidly expanding as MFs could be used as possible building blocks in quantum computation.

In perforated CVD graphene [162], local lattice distortions arising near perforated edges, may induce incredibly large pseudo-magnetic field which will have a sign variation in space [15]. As a result, the obtained zero energy Landau level will be extremely stable [170]. Thus, perforated CVD graphene may be a highly promising candidate material to host MFs even at room temperature. To confirm this premise, recent observations of photoluminescence (PL) in perforated CVD graphene have shown three principal broad lines [162]. We may speculate that the discovered broad band PL emissions can be attributed to the optical transitions between numerous Landau levels induced by fluctuating pseudo-magnetic fields. These findings highlight the importance of considering graphene shape and deformation for its electrical, magnetic and optical properties. Specifically, conducting Raman studies in a high magnetic field presents the next challenge for epitaxial graphene. This opens the door for future Raman spectroscopy investigations into strained graphene and other related 2D materials where the induced effects of pseudo-magnetic fields could be studied.

## 6. Conclusions

In this review, we demonstrate the structural and electrical properties of the SiC substrate. Also, we describe precisely epitaxial graphene growth for both terminations—silicon and carbon—from the SiC substrate. We introduce the Raman analysis of SiC substrate and graphene. We demonstrate different Raman spectroscopy methods for determining the number of graphene layers. The electrical properties of various epitaxial graphene systems are discussed. The density of charge in epitaxial graphene systems of different shapes and layers number is obtained. The studies were done using Raman modes in graphene, particularly focusing on the behavior (I_2D_/I_G_) ratio to determine graphene layer numbers. The obtained results agreed with earlier electrical measurements. Additionally, a new method based on the substrates longitudinal optical phonons (LOOPC) mode has been used to establish the density of charge in epitaxial graphene for different layer shape and numbers have been examined. We have found that the substrate self-compensates the graphene layer charge without any external doping. The new density of charge at the substrate–graphene layer interface emerges as a capacitor that is dominated by the graphene quantum capacitance effect.

## Figures and Tables

**Table 1:** Intensity ratio (I_G_/I_2D_), full width at half maximum (FWHM) of the 2D mode and Raman frequency of the 2D mode of various layer number of epitaxial graphene n_EG_.

Layer Numbers of Epitaxial Graphene n_EG_		Wavelength	I_G_/I_2D_	FWHM of 2D Band or G’ Band	Raman Shift Position of 2D Band
**N = 1**	Ordinary single layer [103]	632.8 nm	<0.5	≈45 cm^−1^	[2655–2665 cm^−1^]
	Bubble of single layer graphene [16]	488 nm	<0.5	[38–63 cm^−1^]	[2721–2724 cm^−1^]
**N = 2**	Ordinary bilayer [104]	532 nm	–	95 cm^−1^	2736 cm^−1^
	Free standing bilayer grapheme [106]	532 nm	–	45–65 cm^−1^	2727 cm^−1^
**N = 4**	Four layers [107]	488 nm	1.5	[87–94 cm^−1^]	[2732–2716 cm^−1^]

FWHM: Full width at half maximum.
